# Proteomic Analysis of Retinas from Two Different Type-1 Diabetic Models

**DOI:** 10.3390/biom16091314

**Published:** 2026-09-10

**Authors:** Gieth Alahdab, Sonali Sharma, Nandini Koneru, Mohamed Moustafa, Muhammad N. Haque, Kaitlin Lowran, Xiao Zhang, Shether Ahmed, Khaled Elmasry, Mohamed Al-Shabrawey

**Affiliations:** 1 Eye Research Center, Oakland University William Beaumont School of Medicine (OUWB-SOM), Rochester, MI 48309, USA; giethalahdab@oakland.edu (G.A.); sonalisharma@oakland.edu (S.S.); nandini.koneru@gmail.com (N.K.); mmoustafa@oakland.edu (M.M.); mnhaque@oakland.edu (M.N.H.); zhang23@oakland.edu (X.Z.); ahmed12@oakland.edu (S.A.); 2 Eye Research Institute, Oakland University, Rochester, MI 48309, USA; 3 Institute of Environmental Health Sciences, Wayne State University, Detroit, MI 48202, USA; hx9909@wayne.edu; 4 Department of Biomedical Sciences, College of Medicine, Dubai Medical University, Dubai P.O. Box 20170, United Arab Emirates; elmasryretina@gmail.com; 5 Foundational Medical Studies, Oakland University William Beaumont School of Medicine (OUWB-SOM), Rochester, MI 48309, USA

**Keywords:** diabetic retinopathy, akita, STZ, type-1 diabetes mellitus, proteome, spectrometry

## Abstract

This study investigated retinal proteomic alterations associated with type-1 diabetes mellitus (T1DM) using two mouse models of diabetic retinopathy (DR): the genetic Ins2^akita/+^ (Akita) model and streptozotocin (STZ)-induced diabetes. Retinas were collected from Akita (n = 4) and STZ-induced diabetic mice (n = 6) 15–16 weeks after the onset of diabetes and compared with age-matched controls. Quantitative mass spectrometry identified 7933 proteins in Akita retinas and 7399 proteins in STZ retinas. Differentially expressed proteins were identified using adjusted *p*-values and log_2_ fold-change, ranked by Manhattan distance, and visualized with volcano plots and heatmaps. The top 20 dysregulated proteins in each model were subjected to canonical pathway analysis. Both models demonstrated upregulation of inflammatory and angiogenesis-associated proteins, including LRRC58, coronin-2A, S100-A4, and COL4A2, supporting a pro-inflammatory and vascular remodeling microenvironment. However, several protein changes differed between the two models. For example, Crystallins were downregulated in the STZ model but upregulated in the Akita model, which were validated by western blot analysis. Canonical pathway analysis revealed activation of platelet-related signaling pathways, enrichment of lipid metabolic networks, and significant alterations in extracellular matrix organization. These findings indicate coordinated inflammatory, metabolic, and structural remodeling in DR and identify candidate molecular pathways for further investigation and therapeutic targeting.

## 1. Introduction

Diabetic retinopathy (DR) is a significant microvascular complication of diabetes mellitus (DM) and continues to be one of the primary causes of vision impairment and blindness in the contemporary world [1,2,3,4]. The rising rates of DR across the world and the resulting burden are expected to increase substantially in the next few decades. DR was estimated at 103 million cases in 2020, and cases are predicted to increase to an estimated 130 and 161 million by 2030 and 2045, respectively [5]. About 4.1 million adult individuals are diagnosed with DR in the United States alone, and one out of every 12 adults over the age of 40 years has an advanced, vision-threatening stage of this disease [6]. In addition to its ocular manifestations, DR serves as an indicator of systemic vascular dysfunction and is associated with increased morbidity and mortality due to its close relationship with the broader vascular complications of diabetes [2].

The pathogenesis of DR is a multifactorial chronic disorder that advances in the sequence of non-proliferative (NPDR), characterized by breakdown of the blood retinal barrier (BRB) and diabetic macular edema (DME), followed by proliferative DR (PDR), defined by abnormal blood vessel growth (retinal neovascularization or RNV) [7]. DME can occur at any stage of DR, though it is more frequently observed in advanced stages. The underlying microvascular injury in DR results from hyperglycemia-induced activation of multiple signaling pathways, notably the upregulation of vascular endothelial growth factor (VEGF) and NF-κB-mediated inflammatory cascades, leading to breakdown of the BRB and subsequent vascular leakage [8]. Clinically, these processes manifest as DME, microaneurysms, intraretinal hemorrhages, cotton–wool spots, intraretinal microvascular abnormalities, venous caliber changes, and neovascularization. These pathological changes can result in significant vision loss through mechanisms, including vitreous hemorrhage, retinal detachment, DME, and retinal capillary nonperfusion [9,10].

Current therapeutic interventions, such as laser photocoagulation and intravitreal pharmacologic therapy (e.g., anti-VEGF and triamcinolone), are limited by significant side effects [11]. These side effects may include long-term postoperative hemorrhage, recurrent retinal detachment, future development of epiretinal membrane after vitrectomy, central retinal vessel occlusion, and neovascular glaucoma [12]. Such limitations highlight the importance of elucidating the molecular pathways involved in the pathogenesis of DR and exploring innovative therapeutic strategies aimed at improving clinical outcomes.

To study DR, a range of experimental diabetic models is widely used. For example, the Akita mouse model (Ins2^akita/+^), which carries a genetic defect causing impaired insulin secretion, and the streptozotocin (STZ)-induced mouse model displaying insulin deficiency due to destruction of pancreatic ß-cells. Both serve as established models for type 1 diabetes mellitus (T1DM). On the other hand, the db/db mouse and Goto-Kakizaki (GK) rat represent experimental type 2 diabetes mellitus (T2DM) models. Since DR is more common in T1DM [13], this study is carried out in experimental models of T1DM utilizing both the Akita mouse and the STZ-induced model.

Although retinal proteomic analyses have previously been separately reported in the Akita and STZ models [14,15], the molecular mechanisms underlying retinal injury in both type-1 diabetic models remain incompletely understood. Furthermore, advances in high-resolution mass spectrometry now enable more comprehensive characterization of the retinal proteome than was previously possible. Therefore, this study was designed as an exploratory, discovery-based proteomic analysis to comprehensively identify proteins and biological pathways associated with diabetic retinopathy in each model. By generating an extensive retinal proteomic dataset, followed by pathway enrichment analysis and validation of selected protein targets, this study provides additional biological insights into the molecular mechanisms of diabetic retinopathy and identifies candidate proteins and pathways that warrant further mechanistic investigation as potential therapeutic targets.

## 2. Materials and Methods

### 2.1. Experimental Mice

Animal studies were carried out at the Eye Research Institute, Oakland University (Rochester, MI, USA) animal facility. The animal protocols were approved by the Institutional Animal Care and Use Committee (IACUC) (Protocol #2022-1159). Akita mice with C57BL/6J background (C57BL/6J Ins2^Akita^) were obtained from Jackson Laboratories (Bar Harbor, ME, USA) [16] and were used in this study as a genetic model of T1DM. Akita mice are characterized by a spontaneous mutation in the insulin 2 gene that leads to incorrect folding of the insulin protein and reduced insulin secretion. Heterozygous Ins2^Akita^ mice develop insulin-dependent diabetes by 3–4 weeks of age and demonstrate early retinal complications such as vascular permeability after 12 weeks and acellular capillaries after 36 weeks [17]. Since the phenotype is more severe in males than females, we only used male Akita in this study and age-matched male C57BL/6J mice as a control group. Genotypes were confirmed according to the Jackson Animal Laboratory genotyping protocols specific to each genotype. Akita mice were diagnosed with diabetes after 2 weeks of birth. Akita and wild-type littermate eyeballs were collected at ~16 weeks post-diabetes. For streptozotocin (STZ)-induced diabetes type 1, 6- to 8-week-old wild-type mice were kept fasting for at least 4 h before intraperitoneal *(IP)* injections of freshly prepared STZ (50 mg/kg, Sigma, St. Louis, MO, USA) dissolved in citrate buffer (Thermo Fisher Scientific, Miller Avenue, Ann Arbor, MI, USA, catalog number 005000, pH 6.0). STZ was administered once daily for 5 consecutive days. Control mice received intraperitoneal injections of an equivalent volume of citrate buffer following the same fasting condition and injection schedule [18]. Mice with blood glucose levels >300 mg/dL were considered diabetic. Only male animals were used in this study. Eyeballs from STZ-injected and their wild-type littermate were collected at 16 weeks post-diabetes. Their retinas were isolated (n = 4 Akita, n = 4 wild-type littermate, n = 6 STZ, and n = 5 wild-type littermate) and snap frozen in liquid nitrogen and stored at −80 °C. Detailed physical and physiological parameters, including sample size, age at termination, initial and final body weight, and fasting blood glucose levels, are summarized in Table 1.

All mice were group-housed, subjected to the standard 12 h light/12 h dark cycle, provided with food and water ad libitum, and kept at a temperature range of 22–24 °C.

### 2.2. Sample Preparation

Mouse retinal samples were homogenized in 100 µL of 20 mM Tris–HCl (pH 8.0) with three 2.0 mm zirconia beads (BioSpec Products, Bartlesville, OK, USA, Cat. No. 11079124zx) using a Storm 24 Bullet Blender (Next Advance, Troy, NY, USA) at setting 8 for 1 min. Following the first round of homogenization, 100 µL of 5% lithium dodecyl sulfate (LiDS) were added to each sample, and homogenization was repeated. Homogenized samples in 2.5% LiDS were heated to 95 °C for 5 min. An aliquot of each lysate was taken for BCA protein analysis, then the remainder of each sample was reduced with 5 mM of DL- dithiothreitol (DTT, Sigma cat# D5545) for 30 min at 37 °C, alkylated with 15 mM of iodoacetamide (IAA, Sigma cat# I1149) for 30 min at room temperature in the dark. Then, 5mM of DTT were added to stop alkylation. Samples were acidified by the addition of 20 µL of 12% phosphoric acid, then proteins were precipitated by the addition of 1 mL of 90% MeOH in 100 mM of TEAB. Pellets from the precipitation were washed with 0.5 mL of 80% MeOH in 10 mM of TEAB. Precipitates were dried on the bench, then resuspended in 25 µL of 20 mM of Tris pH 8.0, 10 mM of CaCl_2_, and 10% Acetonitrile (ACN). Trypsin (Promega, Madison, WI, USA; Cat. No. V5113) was added to each sample, 1 μg per sample, and then incubated overnight at 37 °C to complete the digestion. Peptides were cleaned up by solid-phase extraction on an Oasis HLB 30 mg cartridge (Waters, Milford, MA, USA, Cat. No. WAT094225), dried, and finally reconstituted in 0.1% formic acid before LC-MS analysis.

### 2.3. Mass Spectrometry Analysis

LC-MS/MS analysis was completed using a Thermo Scientific Vanquish-Neo chromatography system with an Acclaim PepMap 100 trap column (100 µm × 2 cm, C18, 5 µm, 100 Å), and an IonOpticks (Melbourne, Australia) Aurora column (75 µm × 25 cm) maintained at 45 °C in an Easy Spray source. A 120 min gradient was applied, starting with 1% solution of B (80% ACN, 0.1% FA), and increasing to the final 42% of solution B. Data-independent analysis (DIA) was performed on a Thermo Scientific Orbitrap Eclipse mass spectrometer. MS1 spectra were acquired at 120,000 resolutions in the 350 to 1200 Da mass range with an AGC of 3e6. MS2 spectra were acquired in the Orbitrap and collected at 15,000 resolutions with a 200-to-1600 Da window. Fragmentation for MS2 spectra was completed using 15 Da windows between 350 and 900 Da with HCD fragmentation at a collision energy of 32, a maximum injection time of 50 msec, and an AGC target of 3e6.

### 2.4. Protein Identification and Quantification

Spectronaut 19.3 (Biognosys AG, Schlieren, Switzerland), using BGS factory settings normalization, and Mouse Uniprot FASTA database (UP000000589, downloaded 30 March 2021), processed mass spectrometry data. The search parameters included trypsin with up to two missed cleavages. Variable modification by oxidation of M and of protein N-termini by Acetylation, Met Loss, or both Acetylation and Met Loss. Carbamidomethylation of cysteine was a fixed modification. For the entire dataset, false discovery rate (FDR) was calculated using a cut-off of 1% for the identification of precursors and 1% for the identification of peptides [19].

### 2.5. Data Analysis

Output data from Spectronaut were imported into VolcaNoseR to generate volcano plots. Differentially expressed proteins were identified based on a log_2_ fold-change threshold ranging from −0.6 to 0.6, and a significance threshold of −Log_10_ *p* =1.8. Proteins meeting these criteria were ranked using Manhattan distance, which integrates effect size and statistical significance.

Heatmaps were generated in R from log_2_-transformed protein intensity values to visualize relative protein abundance patterns across samples for the top 20 differentially expressed proteins. Protein intensities were standardized using z-score transformation across biological replicates. Unsupervised bidirectional hierarchical clustering was performed using Manhattan distance as the dissimilarity metric and complete linkage as the agglomerative clustering method to cluster both sample groups and candidate proteins. Relative expression levels are represented on a standardized color scale, ranging from relative downregulation (blue, z ≤ −1) to relative upregulation (red, z ≥ 2).

Canonical pathway analysis was performed using QIAGEN (Hilden, Germany) Ingenuity Pathway Analysis (IPA), and graphical representations of the top 10 significantly enriched pathways were generated.

### 2.6. Protein Interaction and Functional Enrichment Analysis

Differentially expressed proteins meeting selection criteria *p* < 0.05, q < 0.05, and |AVG Log2 Ratio| ≥ 1 were submitted to the STRING database (v12.0; *Mus musculus*) [20] to construct protein–protein interaction (PPI) networks (7 proteins for STZ vs. control; 12 for Akita vs. control). Interactions were retrieved using a medium confidence threshold score of 0.4.

Functional enrichment analysis was conducted using STRING’s “Values/Ranks” tool (version 12), mapping all quantified proteins and their fold-change values against Gene Ontology (GO: BP, MF, CC), Reactome, and KEGG databases. Statistical significance was calculated using the Benjamini–Hochberg method, with an FDR q < 0.05 cutoff for significance. Top enriched terms were visualized as lollipop plots displaying the enrichment score (*x*-axis), gene count (bubble size), and FDR significance (color gradient).

### 2.7. Western Blot

Tissues were homogenized in urea lysis buffer to extract total protein. Proteins were mixed with Laemmli SDS sample buffer, denatured by heating, and separated on a polyacrylamide gel at 110 V. Proteins were transferred onto a PVDF membrane and blocked with EveryBlot Blocking Buffer (Bio-Rad, Hercules, CA, USA) for 10 min. Membranes were incubated overnight at 4 °C with primary antibodies against βB2 crystallin (1:1000; Invitrogen, Thermo Fisher Scientific, Waltham, MA, USA, cat# PA5-102805), βB3 crystallin (1:1000; Proteintech, Rosemont, IL, USA, cat# 21009-1-AP), and β-actin (1:500; Proteintech, cat# 20535-I-AP), followed by HRP conjugated anti-rabbit secondary antibody (1:500; Cell Signaling Technology, Danvers, MA, USA, cat# 7074S) for 1 h at room temperature. Bands were detected using ECL substrate, imaged with a ChemiDoc Touch system (Bio-Rad, Hercules, CA, USA), and quantified using ImageJ version 1.54.

## 3. Results

**Figure 1:**
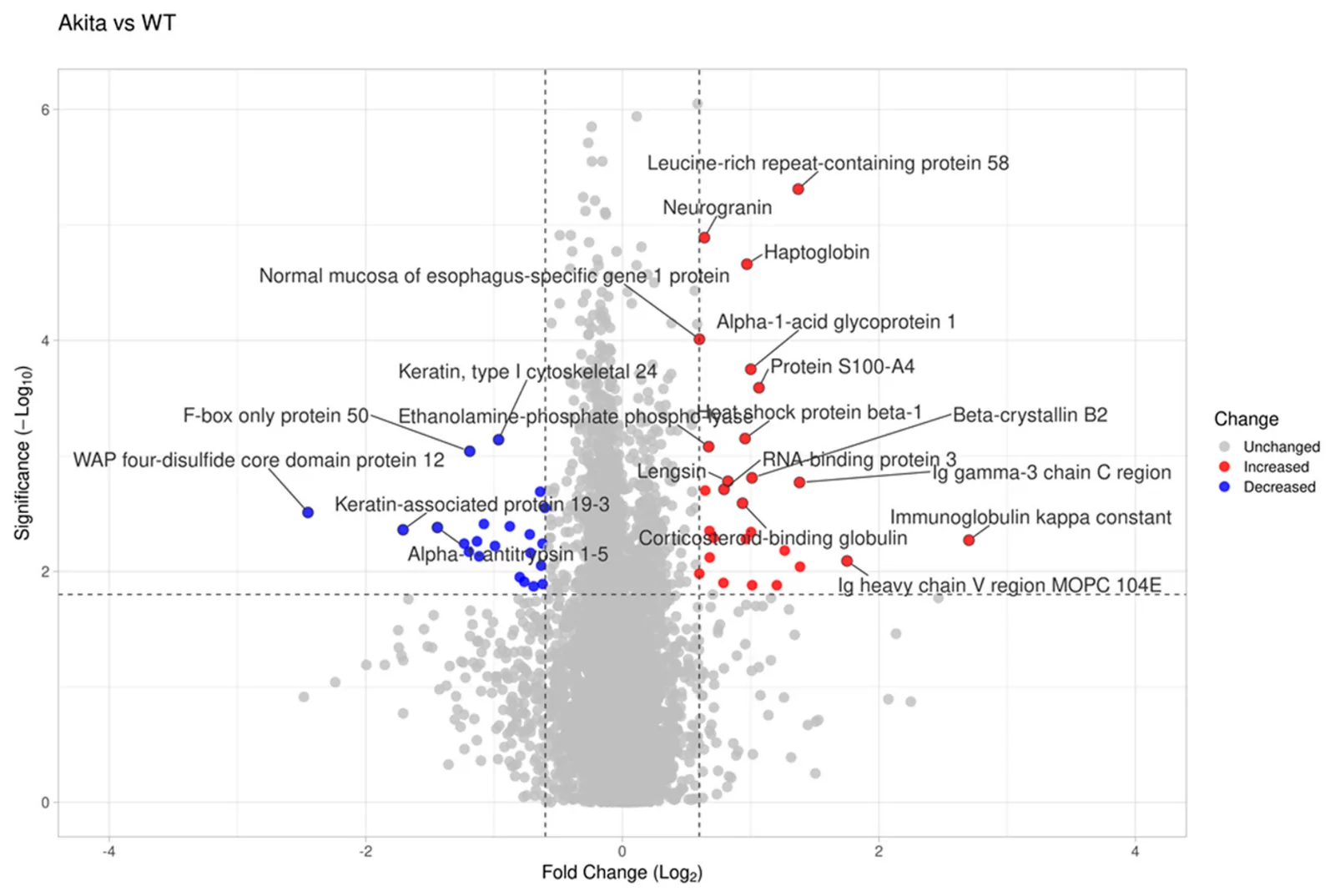
Differential retinal proteome in Akita mice compared with wild-type controls. Volcano plot illustrating differential protein expression in retinas from Akita mice relative to age-matched wild-type (WT) controls. The *x*-axis represents log_2_ fold change, and the *y*-axis represents −log_10_ adjusted *p*-value. Each point corresponds to an identified protein. Vertical dashed lines indicate the predefined log_2_ fold-change threshold, and the horizontal dashed line represents the adjusted *p*-value significance cutoff. Proteins meeting both statistical and fold-change criteria are highlighted in red (increased in Akita) and blue (decreased in Akita), whereas gray points indicate non-significant changes. Selected top 20 dysregulated proteins, determined using Manhattan distance ranking, are annotated.

**Figure 2:**
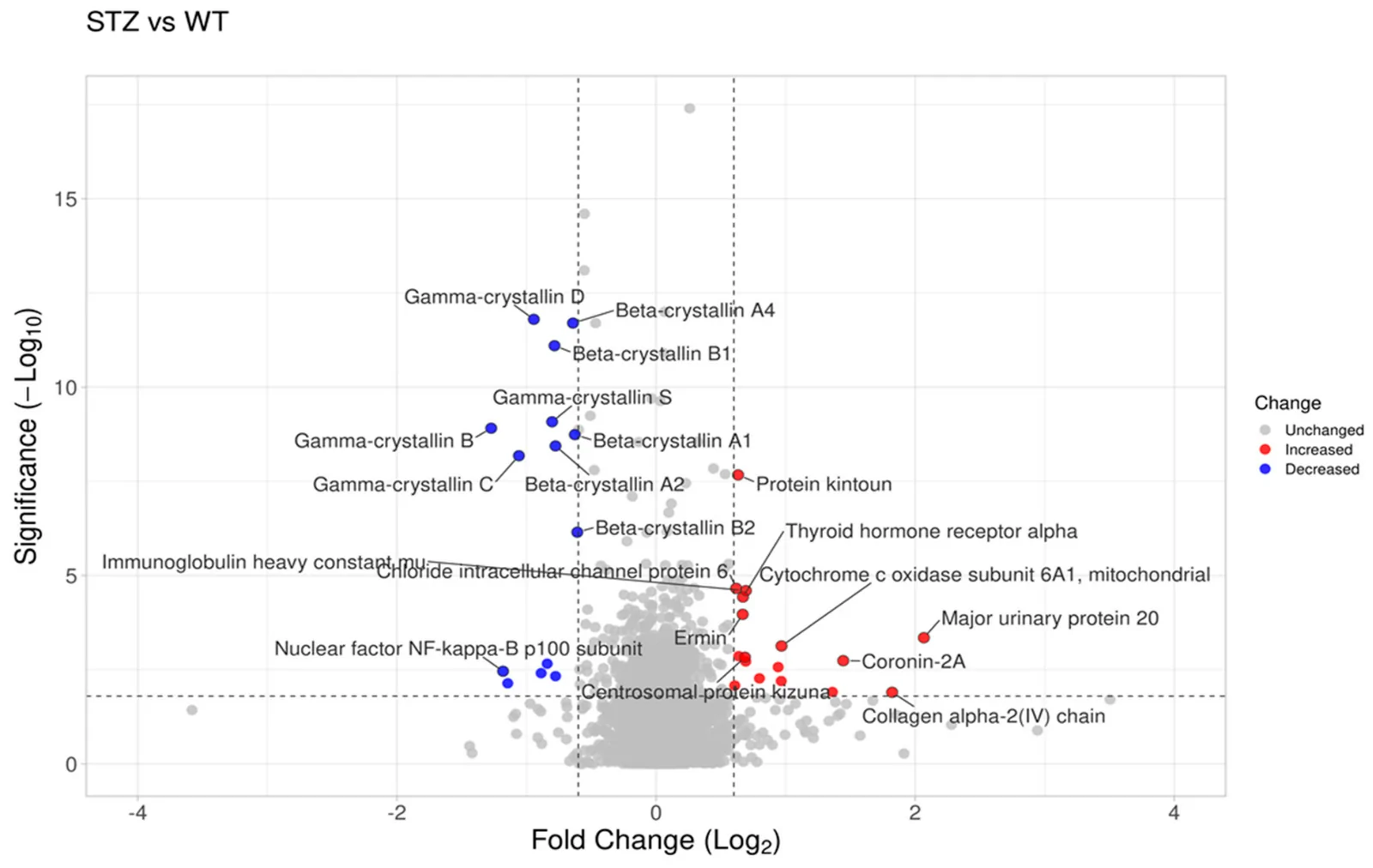
Differential retinal proteome in STZ-treated mice compared with wild-type controls. Volcano plot showing differential protein expression in retinas from STZ-induced diabetic mice relative to age-matched wild-type (WT) controls. The *x*-axis represents log_2_ fold change (STZ/WT), and the *y*-axis represents −log_10_ adjusted *p*-value. Each point corresponds to an identified protein. Vertical dashed lines indicate the log_2_ fold-change threshold, and the horizontal dashed line denotes the statistical significance cutoff. Proteins meeting both criteria are highlighted in red (increased in STZ) and blue (decreased in STZ), while gray points indicate non-significant changes. Selected top 20 dysregulated proteins are annotated.

**Figure 3:**
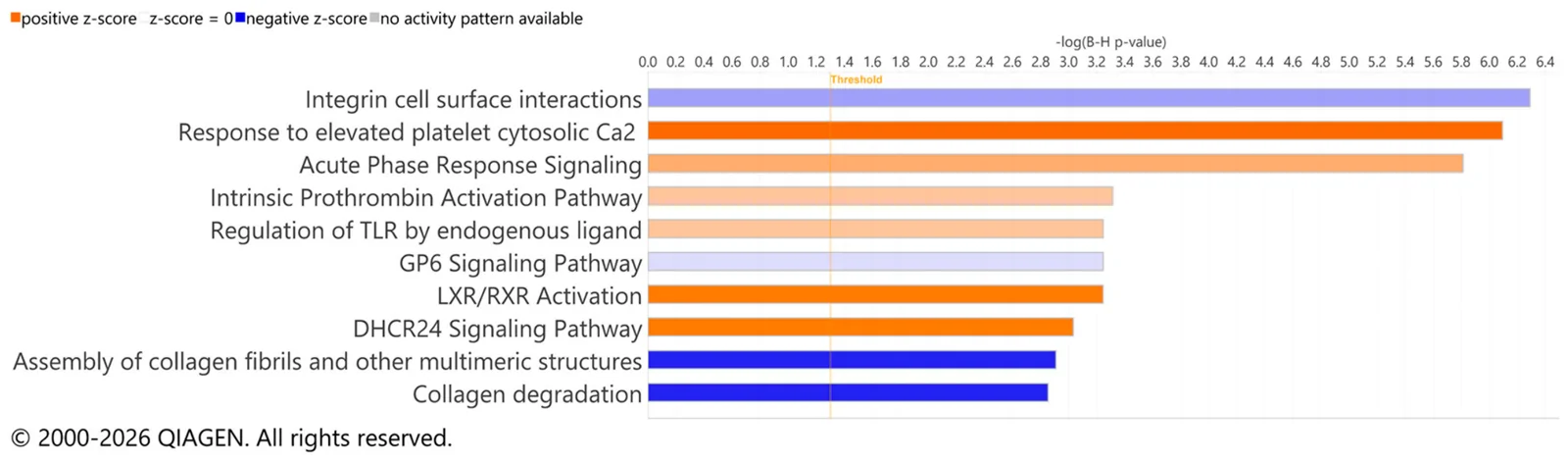
Canonical pathway enrichment analysis in Akita diabetic retinas. Significantly enriched pathways identified by Ingenuity Pathway Analysis (IPA) are shown as −log_10_ (Benjamini–Hochberg adjusted *p*-value). The vertical line indicates the significance threshold. Bar colors represent predicted activity based on z-score: orange, activation; blue, inhibition; gray, no predicted activity pattern.

**Figure 4:**
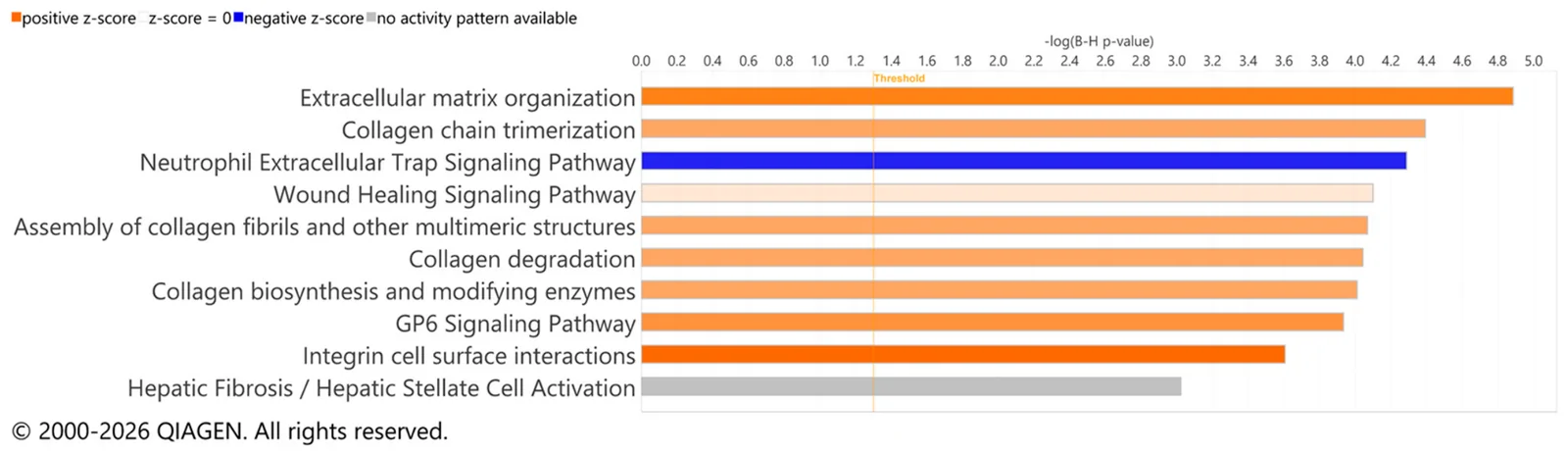
Canonical pathway enrichment analysis in STZ-induced diabetic retinas. Significantly enriched canonical pathways identified by Ingenuity Pathway Analysis (IPA) are shown as −log_10_ (Benjamini–Hochberg adjusted *p*-value). The vertical line indicates the significance threshold. Bar colors denote predicted activity based on z-score: orange, activation; blue, inhibition; gray, no predicted activity pattern.

To characterize proteomic changes linked to DR, we employed data-independent acquisition (DIA)-based mass spectrometry to analyze retinal proteomes from two distinct type-1 diabetes mouse models: the Akita model, representing a genetic form of diabetes, and the streptozotocin (STZ)-induced model, representing a pharmacological form, in comparison with age-matched control mice. Both models were evaluated at 16 weeks following the onset of diabetes. Proteomic analysis performed using Spectronaut (version 19.3) identified 7933 proteins in the Akita group and 7399 proteins in the STZ group. Volcano plots were generated using VolcaNoseR, applying a log_2_ fold-change threshold ranging from −0.6 to 0.6, a significance threshold of −Log_10_ *p* = 1.8, and Manhattan distance as the ranking criterion. The top 20 dysregulated proteins were highlighted in each volcano plot (Figure 1 and Figure 2). To further characterize the most affected biological processes, the top 10 altered canonical pathways (Figure 3 and Figure 4) were identified using QIAGEN Ingenuity Pathway Analysis (IPA). Bar lengths were determined based on Benjamini–Hochberg (B–H) multiple testing–corrected *p*-values, with statistical significance defined as −log_10_(B–H *p*-value) > 1.3. Pathway-activation states were visualized using Z-scores to color the bars.

### 3.1. Top 20 Dysregulated Proteins in Akita Group

**Figure 5:**
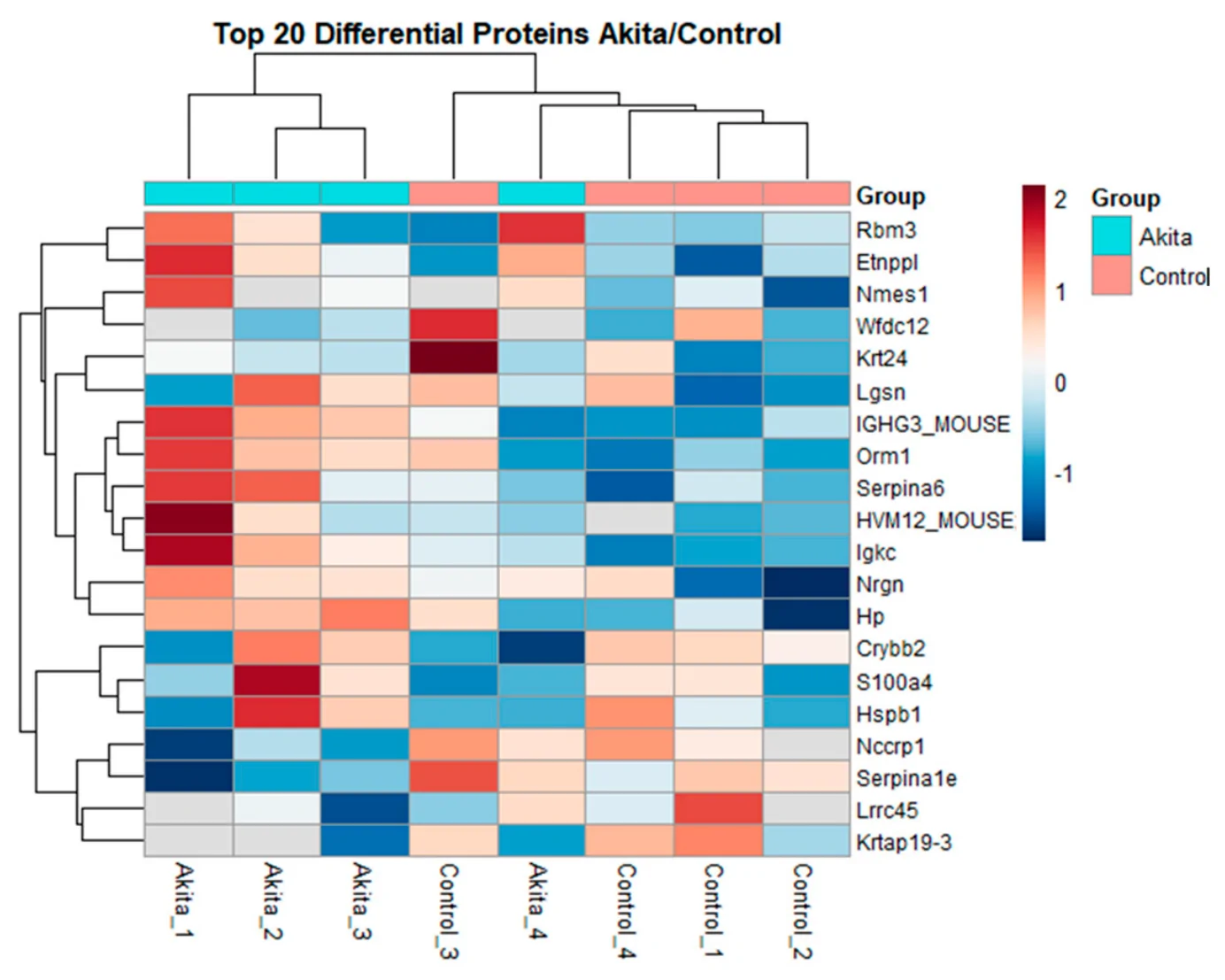
Heatmap of the top 20 differentially expressed proteins in Akita versus control retinas. Hierarchical clustering of the top 20 dysregulated proteins identified by quantitative proteomics. Rows represent proteins and columns represent individual samples. Relative protein abundance is displayed as z-score–normalized expression values (red, increased; blue, decreased).

Among the quantified proteins, the 20 most significantly dysregulated proteins were identified based on volcano plot ranking using Manhattan distance (Figure 1 and Table 2). Of these, 15 proteins were upregulated, and 5 were downregulated in the Akita group compared to its controls. The most significantly increased protein was immunoglobulin kappa constant (IGKC) with a 2.7-fold change (log) and a 2.27 significance (-log_10_), whereas WAP four-disulfide core domain protein 12 showed the greatest decreased protein with a −2.45-fold change (log) and a 2.51 significance (-log_10_). The heatmap was created using the same 20 dysregulated proteins (Figure 5) from the volcano plot. Most Akita samples clustered together and were separated from controls. A subset of proteins showed coordinated upregulation in Akita retinas compared to controls, including immunoglobulin-related proteins (IGHG3_MOUSE, Igkc), acute-phase proteins (Orm1, Hp), and serine protease inhibitors (Serpina6, Serpina1e). Conversely, several proteins were consistently downregulated in Akita samples, including CryβB2 and neuronal-associated proteins.

### 3.2. Canonical Pathway Analysis of Akita Group

Integrin cell surface interactions were the most significantly enriched pathway (6.3 −log *p*-value) (Figure 3). IPA Z-score analysis predicted activation of response to elevated platelet cytosolic Ca2, acute-phase response signaling, intrinsic prothrombin activation pathway, regulation of TLR by endogenous ligand, LXR/RXR activation, and DHCR24 signaling pathway. While IPA Z-score predicted inhibition of Integrin cell surface interactions, GP6-signaling pathway, assembly of collagen fibrils and other multimeric structures, and collagen degradation.

### 3.3. Top 20 Dysregulated Proteins in STZ Group

**Figure 6:**
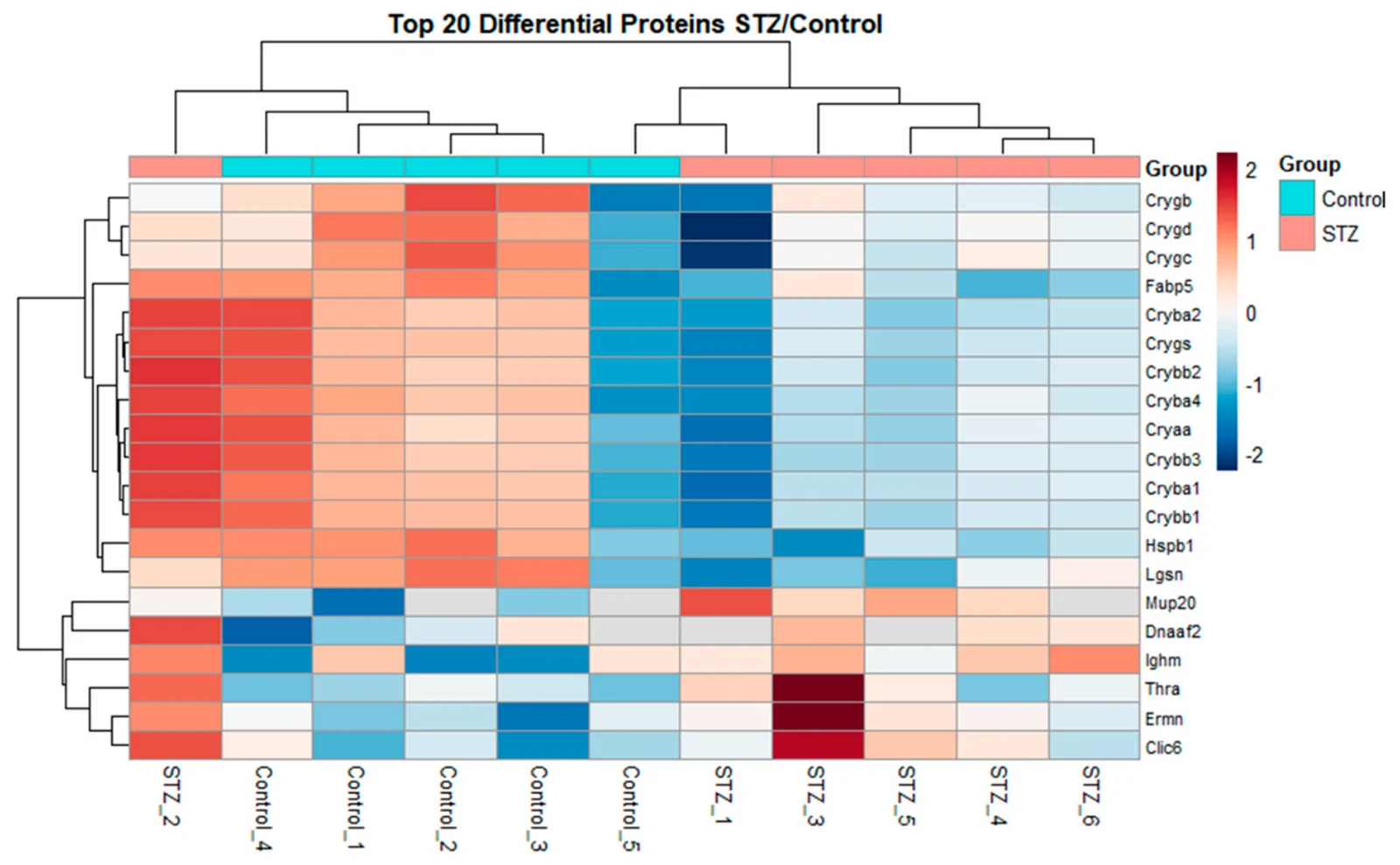
Heatmap of the top 20 differentially expressed proteins in STZ versus control retinas. Hierarchical clustering of the top 20 dysregulated proteins identified by quantitative proteomics. Rows represent proteins and columns represent individual samples. Relative abundance is shown as z-score–normalized expression values (red, increased; blue, decreased).

Among the top 20 dysregulated proteins in STZ group when compared to its controls, 10 of them were upregulated and 10 downregulated (Figure 2 and Table 3). Major urinary protein 20 showed the highest significant fold change increase (2.07-fold change (log) and 3.35 significance (−log^10^)), whereas gamma-crystallin B showed the greatest fold change decrease (−1.27 (log) fold change and 8.91 significance (−log^10^)). Heatmap analysis of the top 20 differentially expressed proteins between STZ and control retinas demonstrated segregation of samples according to experimental group (Figure 6). STZ samples clustered predominantly together and were separated from controls. A coordinated cluster of crystallin family members (including Cryαa, Cryαb isoforms, Cryγs, and CrysβB2) exhibited reduced relative abundance in STZ retinas compared to controls. Additional proteins, including IgHm, Thra, Ermn, and Clic6, also demonstrated group-dependent differential expression.

### 3.4. Canonical Pathway Analysis of STZ Group

The most significantly enriched pathways included extracellular matrix (ECM) organization (4.9 −log *p*-value), collagen chain trimerization (4.4 −log *p*-value), and neutrophil extracellular trap signaling (4.2 −log *p*-value) (Figure 4). IPA Z-score analysis predicted activation of the majority of the top 10 pathways, with neutrophil extracellular trap signaling predicted to be inhibited. Hepatic fibrosis/hepatic stellate cell activation showed no predicted activation state due to the absence of an activity pattern in the IPA database.

### 3.5. PPI Network Analysis

To further investigate the functional relationships among the significantly dysregulated proteins (*p* < 0.05, q < 0.05, and |AVG Log2 Ratio| ≥ 1), STRING network analysis was performed separately on the proteins identified in each experimental group.

**Figure 7:**
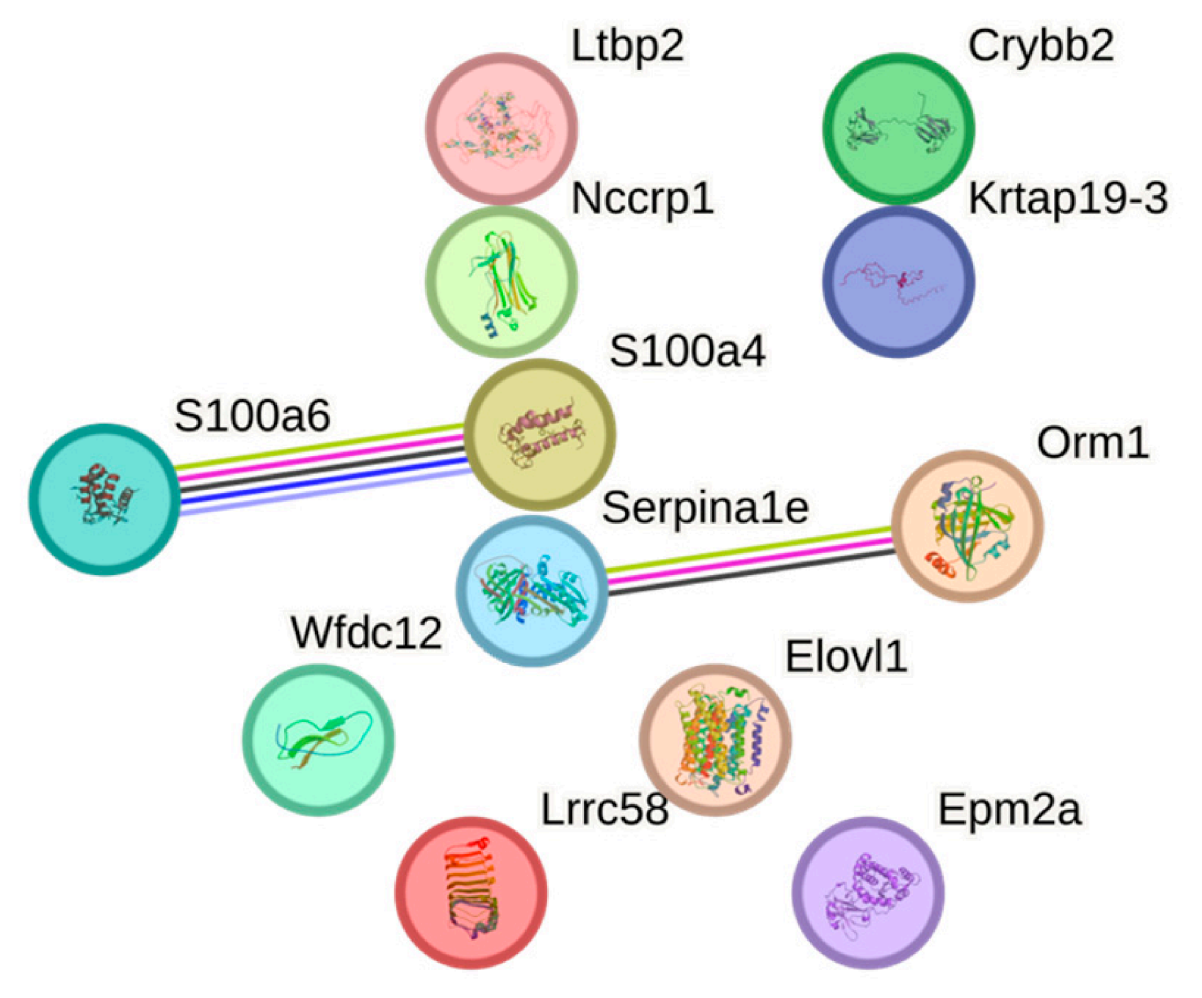
STRING PPI network of dysregulated proteins in the Akita group. Selection criteria for input candidates were *p* < 0.05, q < 0.05, and |AVG Log2 Ratio| ≥ 1. Nodes represent individual proteins with predicted 3D structures, and colored edges denote predicted functional interactions derived from multiple evidence streams (e.g., co-expression, experimental data, and text-mining). The network highlights two key interacting pairs (S100a6–S100a4 and Serpina1e–Orm1), while the remaining unlinked nodes (*Ltbp2*, *Nccrp1*, *Crybb2*, *Krtap19-3*, *Wfdc12*, *Lrrc58*, *Elovl1*, and *Epm2a*) show no predicted interactions at the applied confidence threshold.

In the Akita group (Figure 7), the resulting PPI network mapped 12 out of 14 significant proteins and revealed a sparse interaction topology consisting primarily of isolated nodes, with two distinct interacting protein pairs: S100a6–S100a4: linked by multi-evidence functional interactions (including co-expression and text-mining). Serpina1e–Orm1 exhibits direct predicted interactions associated with acute-phase response and protease regulation. The remaining proteins (*Ltbp2*, *Nccrp1*, *Crybb2*, *Krtap19-3*, *Wfdc12*, *Lrrc58*, *Elovl1*, and *Epm2a*) showed no predicted functional or physical interactions at the specified interaction threshold.

**Figure 8:**
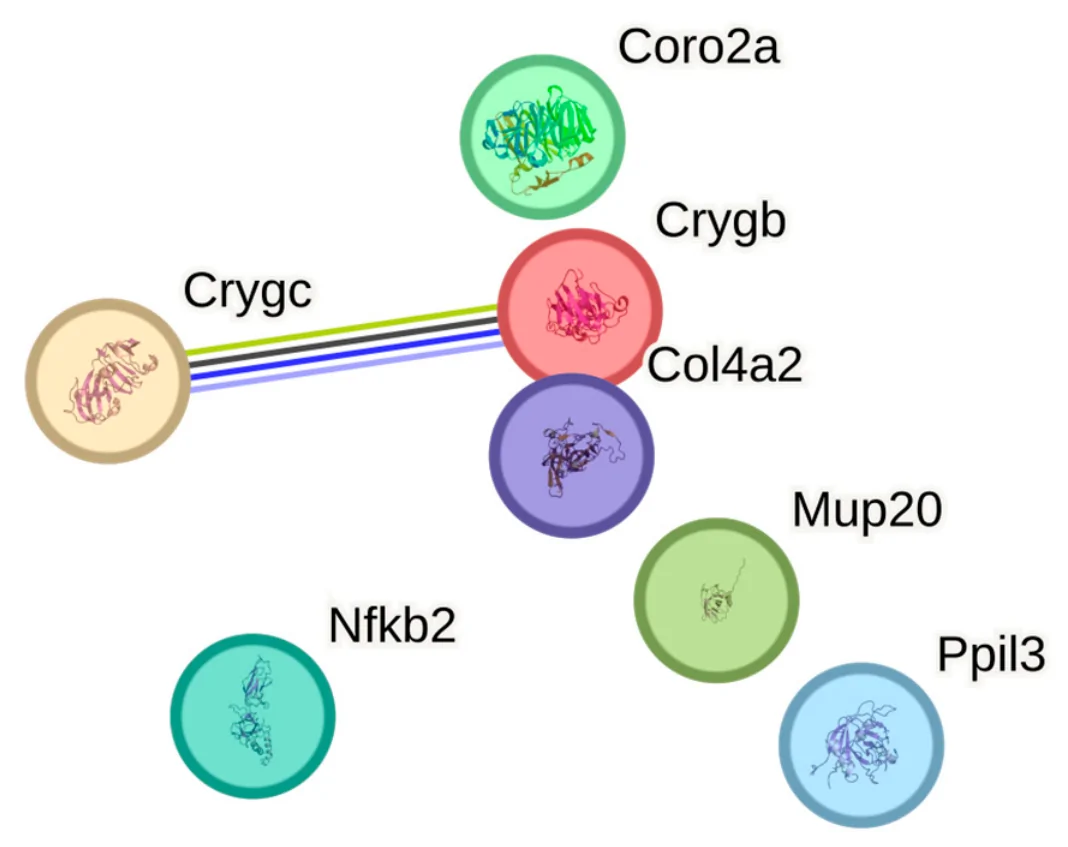
STRING protein–protein interaction (PPI) network of dysregulated proteins in the STZ group. Selection criteria for input candidates *p* < 0.05, q < 0.05, and |AVG Log2 Ratio| ≥ 1. Nodes represent individual proteins with predicted 3D structures, and colored edges denote predicted functional interactions derived from multiple evidence streams (e.g., co-expression, experimental data, and text mining). The network highlights a single interacting pair (Crygc–Crygb), while the remaining unlinked nodes (*Coro2a*, *Col4a2*, *Mup20*, *Nfkb2*, and *Ppil3*) show no predicted interactions at the applied confidence threshold.

Using the same criteria (*p* < 0.05, q < 0.05, and |AVG Log2 Ratio| ≥ 1), STRING network analysis was performed for the STZ group (Figure 8). The resulting PPI network mapped seven out of eight significant proteins and demonstrated a highly disconnected topology, featuring a single functional connection. In the Crygc–Crygb Interaction, a strong functional and structural link was identified between gamma-crystallin C (*Crygc*) and gamma-crystallin B (*Crygb*), reflecting their shared role as major structural components of the eye lens. The remaining five proteins (*Coro2a*, *Col4a2*, *Mup20*, *Nfkb2*, and *Ppil3*) showed no predicted functional or physical interactions at the applied confidence threshold (Supplementary Table S1).

**Figure 9:**
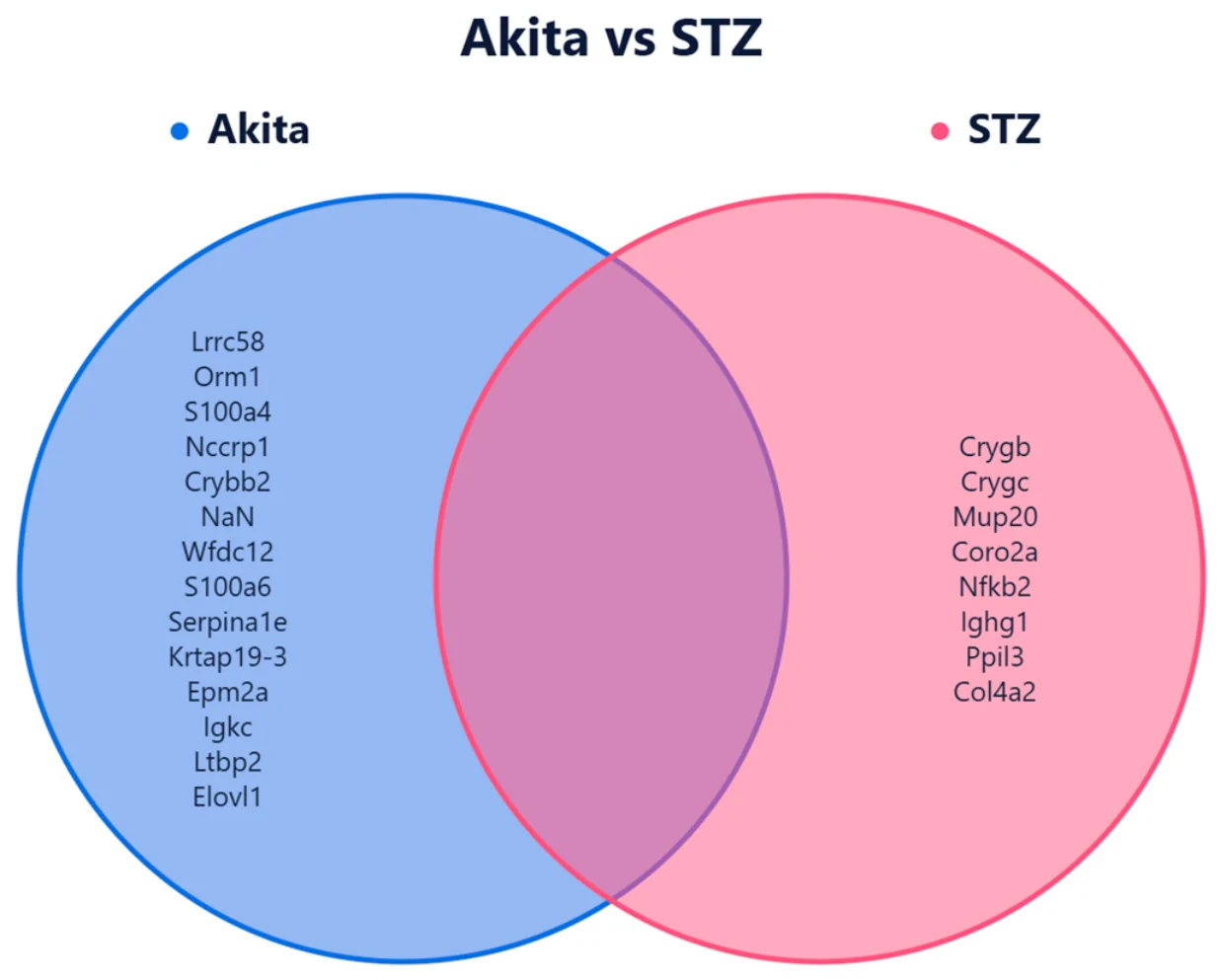
Venn diagram of differentially expressed proteins in Akita vs. STZ retinas. Comparison of significantly dysregulated retinal proteins in Akita (n = 14, blue) and STZ (n = 8, pink) mouse models compared to non-diabetic controls *p* < 0.05, q < 0.05, and |AVG Log2 Ratio| ≥ 1). Zero overlapping proteins were identified between the two models.

No overlapping significantly dysregulated proteins were identified between the Akita (n = 14) and STZ (n = 8) groups (Figure 9).

### 3.6. Functional Enrichment and Pathway Analysis of the Dysregulated Proteins

**Figure 10:**
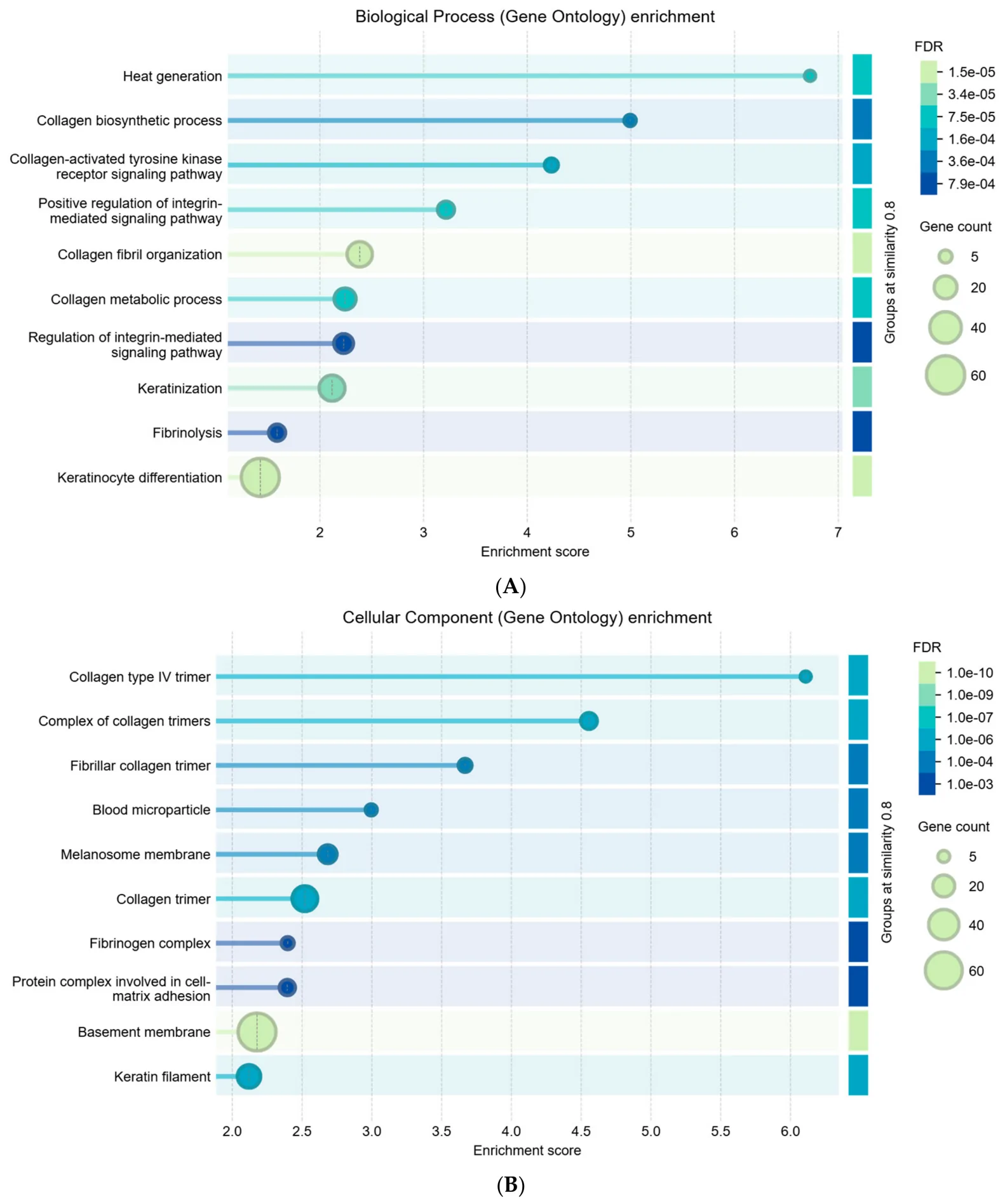

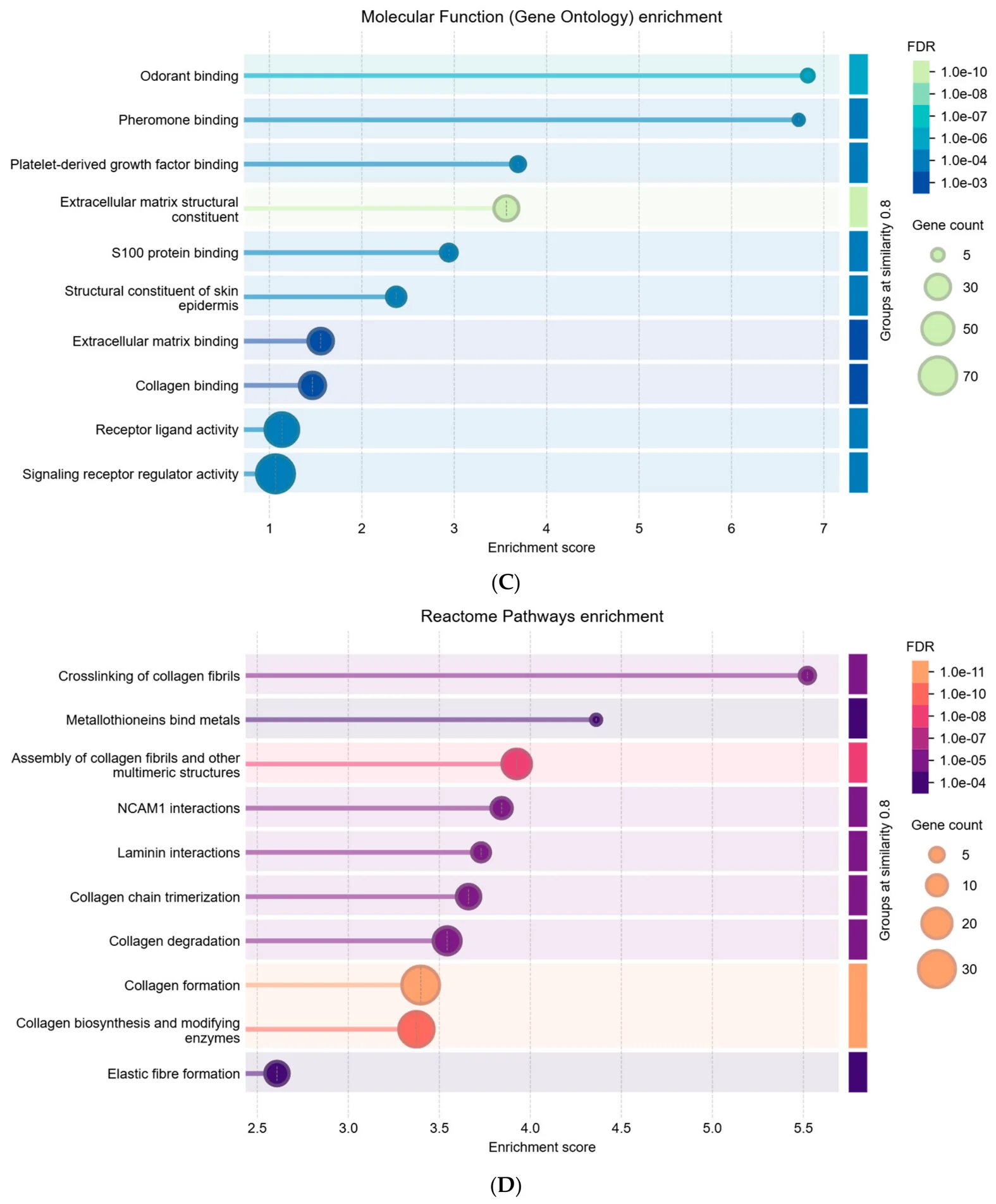
Functional enrichment and pathway analysis of dysregulated proteins in the Akita vs. Control group. Lollipop plots displaying the top enriched terms for (**A**) Biological Process (GO), (**B**) Cellular Component (GO), (**C**) Molecular Function (GO), and (**D**) Reactome Pathways. The horizontal position of each bubble indicates the enrichment score, the bubble size reflects the number of genes associated with the term (Gene count), and the color gradient represents the False Discovery Rate (FDR).

**Figure 11:**
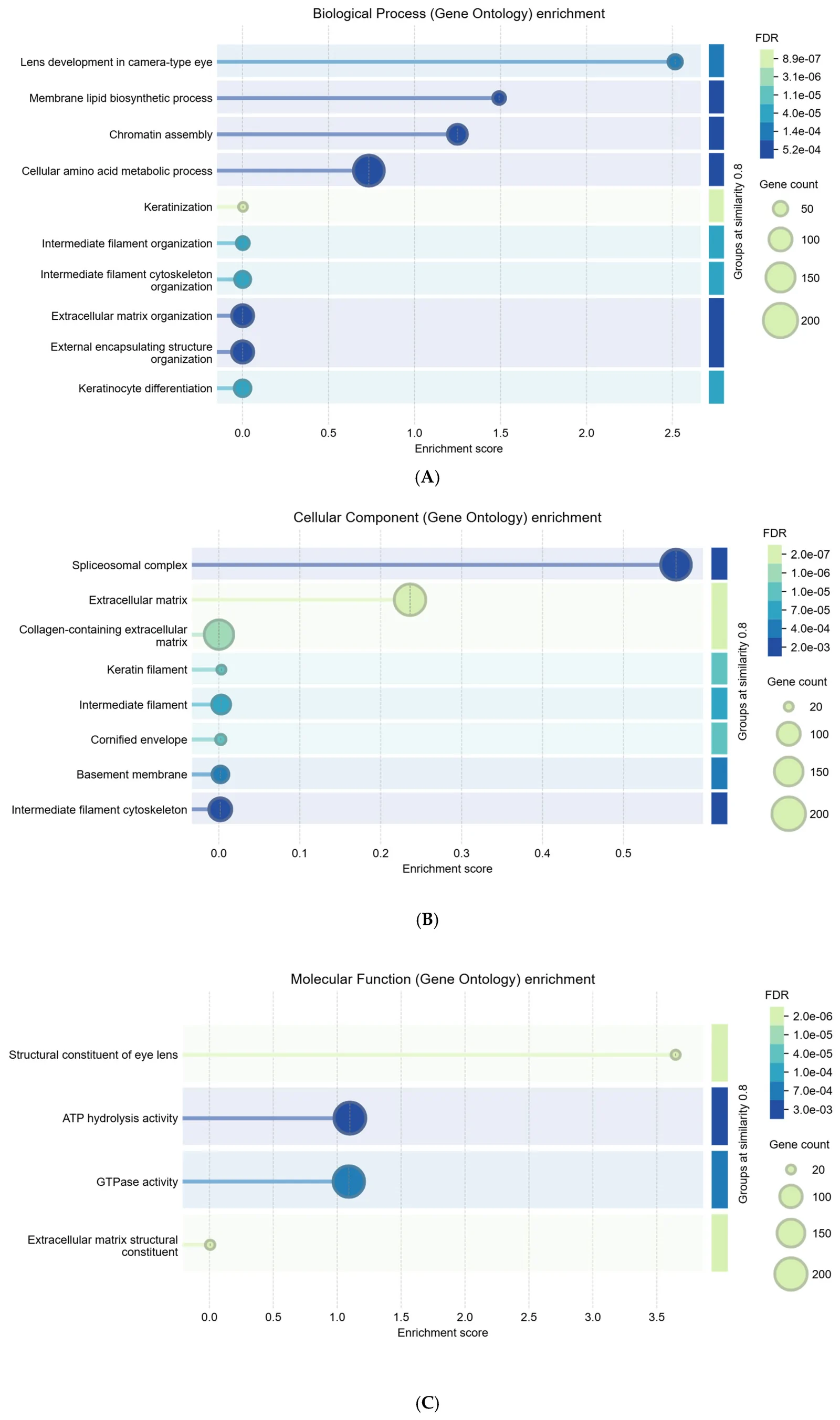

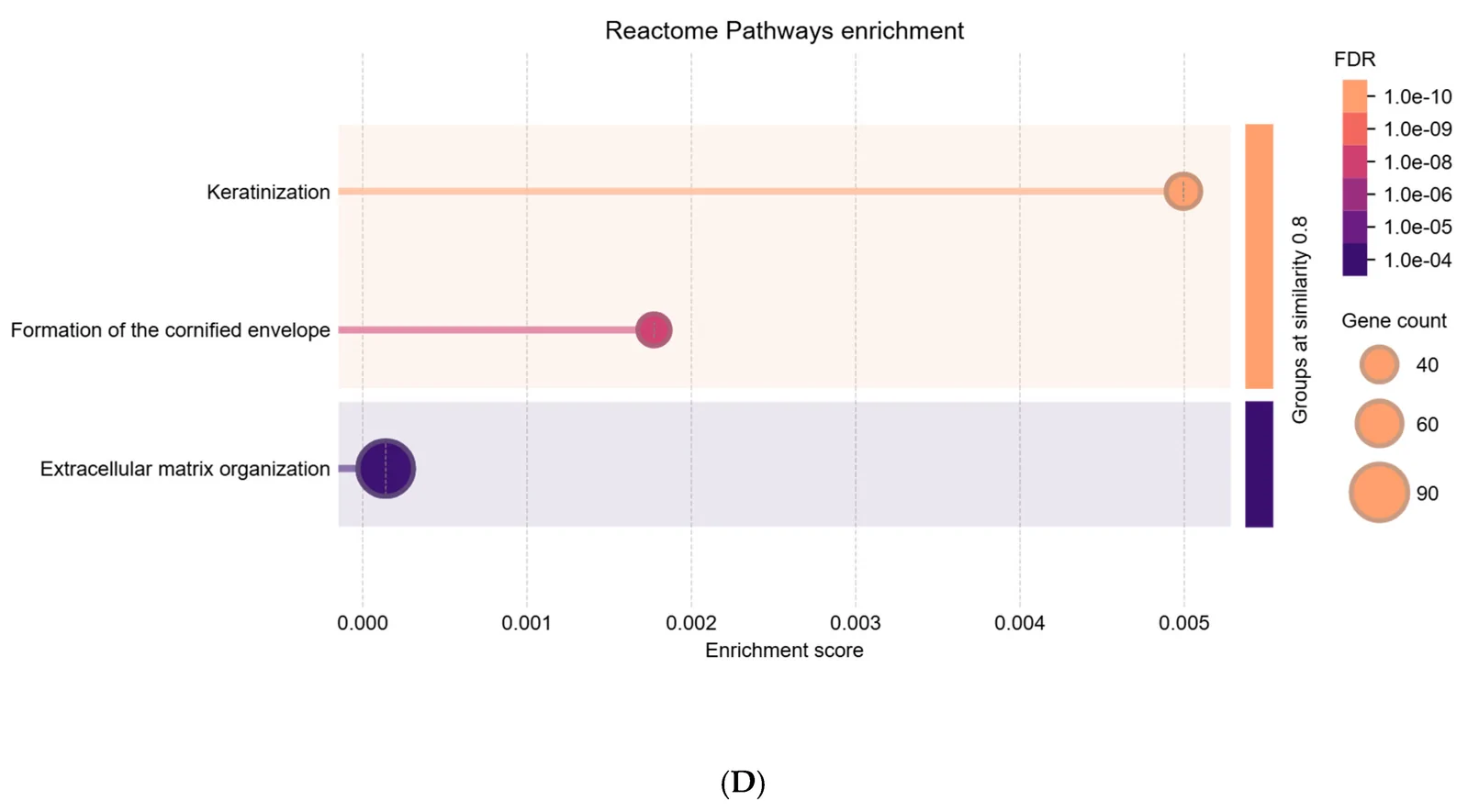
Functional enrichment and pathway analysis in the STZ group. Lollipop plots displaying the top enriched terms for (**A**) Biological Process (GO), (**B**) Cellular Component (GO), (**C**) Molecular Function (GO), and (**D**) Reactome Pathways. The horizontal position of each bubble indicates the enrichment score, the bubble size reflects the number of genes associated with the term (Gene count), and the color gradient represents the False Discovery Rate (FDR).

Gene Ontology (GO)-enrichment analysis was performed using the 7933 proteins in the Akita group (Figure 10) and 7399 proteins in the STZ group (Figure 11).

In the Akita group (Figure 10), functional enrichment across GO categories—Biological Process (BP), Cellular Component (CC), Molecular Function (MF)—and Reactome Pathways revealed extensive remodeling of the ECM and collagen networks: BP: top enriched terms included Heat generation, Collagen biosynthetic process, Collagen-activated tyrosine kinase receptor-signaling pathway, and Positive regulation of integrin-mediated signaling pathway, as well as matrix organization terms such as Collagen fibril organization and Fibrinolysis. CC: The most strongly enriched compartments were centered on collagen architecture, including Collagen type IV trimer (enrichment score > 6), Complex of collagen trimers, Fibrillar collagen trimer, and the Basement membrane FDR < 10^−10^). MF: Overrepresented functions included Odorant binding, Pheromone binding, Platelet-derived growth factor binding, S100 protein binding, and Extracellular matrix structural constituent. Reactome Pathways: Corroborating the GO terms, key enriched pathways included Crosslinking of collagen fibrils, Metallothioneins bind metals, Assembly of collagen fibrils and other multimeric structures, and NCAM1 interactions.

In the STZ group (Figure 11), functional enrichment analysis of the 7399 identified proteins demonstrated distinct pathway and ontology representations across GO categories and Reactome pathways, with notable signatures in ocular development, enzymatic activity, and structural matrix integrity. BP: top enriched biological processes included Lens development in camera-type eye (highest enrichment score), Membrane lipid biosynthetic process, Chromatin assembly, and Cellular amino acid metabolic process. Additional structural terms such as Extracellular matrix organization, Intermediate filament organization, and Keratinization were also enriched. CC: Enriched cellular compartments were prominently led by the Spliceosomal complex (highest enrichment score), followed by structural and extracellular compartments, including Extracellular matrix, Collagen-containing extracellular matrix, Basement membrane, and Intermediate filament cytoskeleton. MF: The most strongly enriched molecular function was Structural constituent of eye lens (enrichment score > 3.5), followed by nucleotide-binding and enzymatic functions such as ATP hydrolysis activity, GTPase activity, and Extracellular matrix structural constituent. Reactome Pathways: Corroborating the GO observations, Reactome pathway analysis highlighted epidermal/structural integrity and matrix remodeling, with Keratinization FDR < 10^−10^) and Formation of the cornified envelope showing the highest statistical significance, alongside Extracellular matrix organization.

### 3.7. Retinal Expression of β B-Crystallins in Akita and STZ Mice

**Figure 12:**
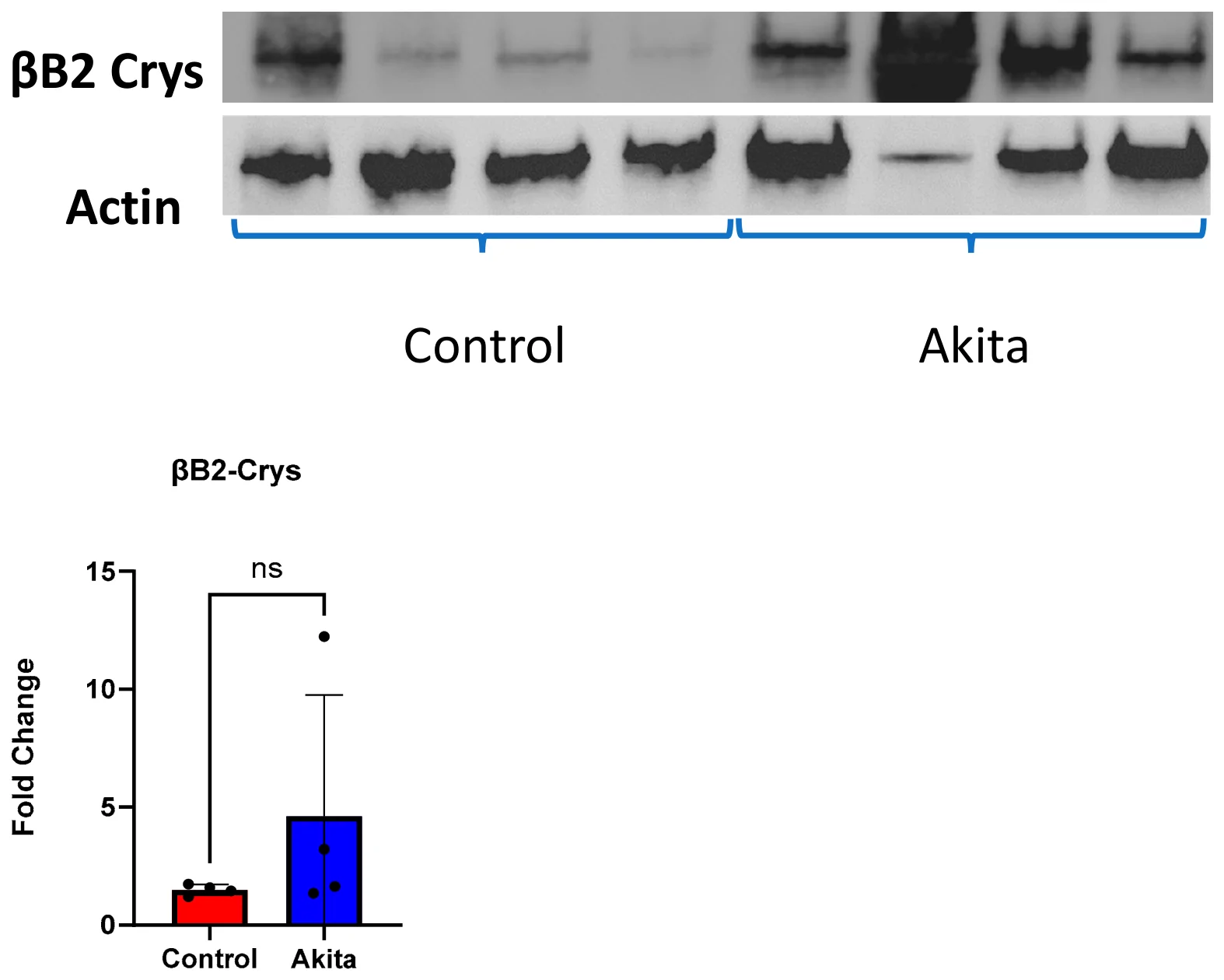
Western blot validation of crystallin expression in control and Akita diabetic retina. Representative Western blots showing protein expression of βB2-crystallin (βB2-Crys) in retinal samples from control and Akita diabetic mice. Actin was used as a loading control. Densitometric quantification of βB2-Crys normalized to actin is shown below. Data are presented as fold change relative to control. βB2-Crys expression showed a non-significant increasing trend in Akita samples compared to control (ns). Data are presented as mean ± SEM, with individual data points shown. The original western blot images can be found at Figure S1.

Using western blot expression of β-crystallin, B2 (βB2-Crys) was measured in both STZ and Akita groups. In the Akita group (Figure 12), βB2-Crys expression exhibited a non-significant increasing trend in the Akita group (Mean ± SD: 4.614 ± 5.147) compared to control (Mean ± SD: 1.5 ± 0.2287).

**Figure 13:**
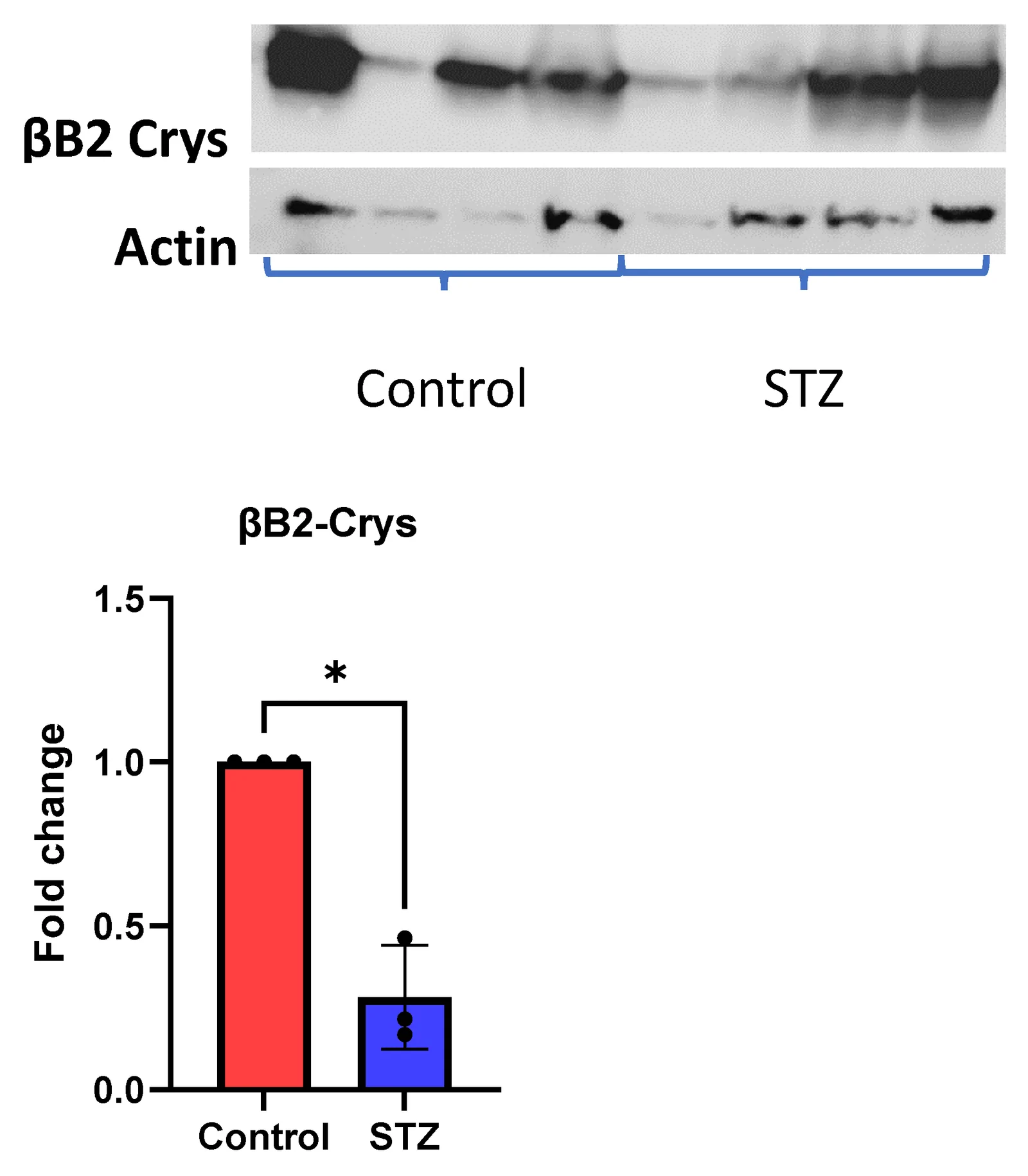
Western blot validation of crystallin expression in control and STZ-induced diabetic retina. Representative Western blots showing protein expression of βB2-crystallin (βB2-Crys) and in retinal samples from control and STZ-induced diabetic mice. Actin was used as a loading control. Plotted values represent the means of three independent experiments compiled from a total of n = 10 Control and n = 8 STZ samples. Bars represent Mean ± SD: 0.2824 ± 0.1579 vs. Control normalized to 1.0; *p* = 0.0158. * means it is significant. The original western blot images can be found at Figure S2.

To determine the impact of STZ treatment on β-crystallin B2 (βB2-Crys) protein levels, western blot analysis was performed using protein extracts collected across three independent experimental cohorts (n = 10 Control, n = 8 STZ). Band intensities were normalized to actin and calculated as relative fold change compared to the control group mean. Quantitative densitometry revealed a significant reduction in βB2-Crys protein expression in the STZ group compared to the Control group (Mean ± SD: 0.2824 ± 0.1579 vs. Control normalized to 1.0; *p* = 0.0158, two-tailed Student’s *t*-test) (Figure 13).

## 4. Discussion

The current study underscores the most dysregulated retinal proteins and related signaling pathways in two T1DM mouse models at ~16 weeks post-diabetes, a key transition period between early and advanced retinal microvascular dysfunction. Importantly, this work provides novel insights by highlighting previously unrecognized therapeutic targets for diabetic retinopathy, with a particular emphasis on crystallins. Among the notable proteomic differences between the Akita and STZ-induced models, a compensatory response was evident in the Akita retina. Specifically, crystallins were upregulated in the Akita model, whereas a downregulation occurred in the STZ model. Such differential regulation may contribute to the delayed onset of vascular and neuronal injury in Akita retinas, offering valuable guidance for both experimental design and model-specific therapeutic interventions. Volcano plot analysis revealed widespread proteomic alterations in the Akita retina. Among the top 20 dysregulated proteins ranked by Manhattan distance—which integrates both fold change and statistical significance—we focus specifically on those with established roles in diabetic retinopathy. Among these, IGKC was the most upregulated protein based on log_2_ fold change. IGKC encodes the constant region of kappa light chains of immunoglobulins, a key component of B-cell–mediated humoral immune responses [21]. In addition to IGKC, immunoglobulin heavy chain V region MOPC 104E and immunoglobulin gamma-3 chain C region (IgG3) were also significantly upregulated in the Akita retina. The concurrent upregulation of immunoglobulin light- and heavy-chain components suggests enhanced humoral immune activity within the diabetic retina [22].

Leucine-rich repeat-containing protein 58 (LRRC58) functions as a substrate adaptor for an E3 ubiquitin ligase complex that targets cysteine dioxygenase 1 (CDO1) [23]. Taurine is highly enriched in the retina and plays a critical role in protecting retinal neurons from oxidative and metabolic stress. Dysregulation of LRRC58 may therefore influence retinal cysteine–taurine metabolism, potentially reducing taurine availability and exacerbating oxidative stress, lipid dysregulation, and inflammatory processes that contribute to diabetic retinopathy [24].

Protein S100-A4 participates in angiogenesis, an immune response, and plays a role in cancer metastasis [25]. S100-A9, a protein from the same family, has been reported to induce neurodegeneration and vascular abnormalities [26]. Therefore, the role of S100-A4 in DR warrants further study, as it represents a compelling candidate for understanding vascular changes in the retina. This notably includes CrysβB2, an abundant lens protein which has been also found to be expressed in retina [27]. In the retina, βB2-Crys plays a protective role by preventing degeneration of the retinal pigment epithelium and has been shown to be upregulated during retinal regeneration, where it promotes retinal ganglion cell survival [28]. CrysβB2 was identified among the top 20 significantly dysregulated proteins in the Akita mouse retina. Given that CrysβB2exerts a protective effect by preventing retinal pigment epithelium degeneration and promoting retinal ganglion cell survival during regeneration [28], its downregulation points to a potential loss of neuroprotective capacity during DR progression. Because the divergent regulation of crystallins represented the most robust and intriguing candidate pathway identified in our proteomic screen, we selected crystallin proteins for downstream independent validation in separate biological replicates. Western blot analysis was performed on independent samples. We assessed the expression of βB2-Crys and (Figure 13). Consistent with the proteomic data, Western blotting demonstrated an increasing trend in βB2-Crys expression. Although this increase did not reach statistical significance, the observed trend was in the same direction as that detected by the proteomic analysis.

Acute-phase proteins alpha-1-acid glycoprotein 1, Haptoglobin, Heat shock protein beta-1, and corticosteroid-binding globulin are typically induced during local and systemic inflammation. Their upregulation is consistent with the chronic inflammation in DR [29,30,31].

Neurogranin is a neuronal calcium-binding protein that regulates calmodulin-dependent signaling and synaptic function. Its dysregulation in the diabetic retina supports the concept that diabetic retinopathy involves early neuronal and synaptic alterations in addition to vascular pathology [32].

Ethanolamine–phosphate phospho-lyase participates in phospholipid metabolism by regulating ethanolamine phosphate turnover, thereby influencing membrane composition and lipid homeostasis [33]. Altered expression of this protein in the diabetic retina may reflect disrupted membrane lipid metabolism—a process implicated in cellular dysfunction and stress responses—making it an intriguing candidate that warrants further investigation.

F-box only protein 50 (FBXO50) is a component of E3 ubiquitin ligase complexes that regulate protein degradation through the ubiquitin–proteasome system [34]. Downregulation of FBXO50 in the diabetic retina may indicate altered proteostasis and adaptive regulation of stress- or inflammation-related proteins in response to chronic metabolic challenge.

Keratin, type I cytoskeletal 24 (KRT24), and keratin-associated protein 19-3 (KRTAP19-3) are structural proteins linked to intermediate filament organization and cytoskeletal stability. Keratins constitute a major component of the cellular cytoskeleton, providing mechanical integrity and participating in stress-adaptive responses, while keratin-associated proteins regulate filament assembly and structural reinforcement [35]. Although the retina is not classically considered a keratin-rich tissue, the altered abundance of these proteins in the diabetic retina may reflect stress-induced cytoskeletal remodeling or changes in cellular phenotype under chronic metabolic challenge.

Alpha-1-antitrypsin proteins are serine protease inhibitors with established anti-inflammatory and tissue-protective properties. Downregulation of alpha-1-antitrypsin isoforms in the diabetic retina may reflect altered protease–antiprotease balance [36].

Whey acidic protein four-disulfide core domain protein 12 (WFDC12) exhibited the greatest decrease in abundance based on log_2_ fold change, indicating marked suppression in the diabetic retina. WFDC12 is associated with protease regulation and immunomodulatory functions; therefore, its downregulation may suggest impaired protease–antiprotease balance or reduced protective regulatory mechanisms within the diabetic retina [37].

Canonical pathway analysis predicted a coordinated shift in signaling networks characterized by activation of inflammatory and stress-associated pathways alongside suppression of ECM regulatory mechanisms. The enrichment of the Acute Phase Response and Toll-like receptors (TLR) regulation strongly supports activation of innate immune and inflammatory processes. Acute phase signaling activation reflects cytokine-driven systemic or tissue-level responses [38], while activation of TLR-associated pathways indicates cellular stress, tissue injury, or chronic inflammation [39].

Activation of platelet cytosolic Ca^2+^ signaling and the intrinsic prothrombin activation pathway suggests involvement of calcium-dependent signaling and coagulation cascade. Calcium flux is central to platelet activation and intracellular signaling [40], and prothrombin pathway enrichment often accompanies vascular stress, endothelial dysfunction, or inflammatory microenvironments [41].

The activation of Liver X Receptor/Retinoid X Receptor (LXR/RXR) and 24-dehydrocholesterol reductase (DHCR24) signaling implies modulation of lipid metabolic and cytoprotective pathways. LXR is tightly linked to lipid metabolism. RXR is involved in cell proliferation and vitamin D metabolism [42].

Activation of the DHCR24-signaling pathway in the diabetic condition may suggest adaptive modulation of sterol metabolism and cytoprotective responses. DHCR24 functions as a key enzyme in cholesterol biosynthesis and has been implicated in cellular resistance to oxidative and metabolic stress. Given that diabetes imposes significant lipid and redox imbalance, enrichment of DHCR24-associated signaling likely represents a compensatory response aimed at maintaining cholesterol homeostasis and protecting against stress-induced cellular dysfunction [43].

Both integrin signaling and GP6 signaling are fundamentally linked to cell–collagen and cell–matrix interactions. GP6 functions as a major collagen receptor, while integrins mediate cell adhesion and bidirectional ECM signaling [44,45]. Their concurrent inhibition strongly suggests reduced matrix sensing and cell–ECM communication. The suppression of collagen assembly and collagen degradation indicates attenuated matrix remodeling dynamics [46]. Taken together, inhibition of integrin and GP6 pathways alongside suppressed collagen turnover supports a model of impaired ECM regulatory activity. This pattern may reflect disrupted structural adaptation and diminished tissue remodeling capacity.

Heatmap analysis of the top differentially expressed proteins in Akita retinas revealed coordinated upregulation of immunoglobulin components, acute-phase reactants, and serine protease inhibitors, supporting activation of inflammatory and humoral immune mechanisms in the diabetic retina. Concurrent upregulation of crystallin family members suggests impairment of intrinsic cytoprotective pathways that normally buffer oxidative and metabolic stress. The clustering of protease-regulatory and structural proteins further indicates remodeling of ECM and barrier-associated processes. Importantly, the modular organization observed in the heatmap suggests network-level proteomic reprogramming rather than isolated molecular perturbations, reinforcing pathway-level alterations identified in canonical pathway analysis.

Major urinary protein 20 (MUP20) showed the largest fold change in the STZ group. MUP20 is a liver-enriched secreted lipocalin that circulates in the bloodstream and is ultimately excreted in urine. Therefore, its marked dysregulation may reflect a broader systemic response to STZ-induced insulin deficiency and metabolic stress rather than a purely retina-intrinsic pathway. Notably, the detection of MUP20 in retinal tissue may be attributable to breakdown of the blood–retinal barrier (BRB), a well-established feature of DR, which permits increased extravasation and accumulation of circulating serum proteins within the retina [47].

Consistent with our proteomic data, which show marked upregulation of Collagen alpha-2(IV) chain (COL4A2) in STZ group, recent evidence indicates that COL4A2 actively promotes endothelial cell proliferation, migration, and angiogenesis via AKT signaling in diabetic retinopathy models. This supports a role for COL4A2 in diabetic vascular remodeling beyond its classical structural function in basement membranes [48].

Cytochrome c oxidase subunit 6A1 (COX6A1) has been implicated in modulation of mitochondrial function and cellular responses to oxidative stress, with evidence suggesting roles in limiting reactive oxygen species (ROS) accumulation and supporting anti-apoptotic mechanisms [49]. Its upregulation in our proteomic may therefore represent a compensatory adaptation of the mitochondrial electron transport chain in response to diabetes-associated impairment of oxidative phosphorylation.

Immunoglobulin heavy constant mu (IGHM) represents the constant region of IgM antibodies. Upregulation indicates immune activation, vascular permeability changes, or inflammatory infiltration [50].

Ermin is an oligodendrocyte/myelin-associated cytoskeletal protein involved in myelin sheath organization [51]. Upregulation may reflect glial responses, cytoskeletal remodeling, or myelin-related adaptations under metabolic stress.

A prominent feature of the downregulated proteins in the STZ group was the coordinated reduction of multiple crystallin family members, including β-crystallins (βB2-Crys, βA1-Crys, βA4-Crys, βA2-Crys, βB1-Crys) and γ-crystallins (CRY-GS, CRY-GD, CRY-GC, CRY-GB). Although classically recognized as structural lens proteins, crystallins are widely expressed in neural tissues where they serve critical cytoprotective and stress-responsive functions, including molecular chaperoning, stabilization of cytoskeletal elements, and protection against oxidative damage [52]. Decreased crystallin abundance has been repeatedly associated with cellular vulnerability, impaired proteostasis, and reduced resistance to metabolic and oxidative stress [53]. In particular, β-crystallins have been implicated in neuronal survival and cytoskeletal maintenance, while γ-crystallins contribute to protein stability and stress tolerance. Therefore, the observed crystallin downregulation may reflect compromised endogenous protective mechanisms under diabetic conditions, potentially exacerbating oxidative injury and cellular dysfunction.

To validate our proteomic findings, Western blot analysis of βB2-Crys was performed using independent samples collected across three separate experimental cohorts (Figure 13). Consistent with the proteomic data, Western blotting confirmed a significant down-regulation in βB2-Crys protein expression in the STZ group.

Additionally, the reduction of the NF-κB p100 subunit (NFKB2) suggests perturbation of NF-κB-signaling dynamics. The p100 precursor functions both as an inhibitor and a source of the active p52 transcription factor in the non-canonical NF-κB pathway, which regulates inflammatory responses, cell survival, and stress adaptation [54]. Downregulation of p100 may indicate altered inflammatory regulation or impaired stress-responsive transcriptional control in the diabetic environment.

The pathway analysis revealed a dominant enrichment of ECM and collagen-related processes, accompanied by signatures of inflammatory and platelet-associated signaling, suggesting that STZ-induced diabetes drives coordinated structural and stress-response remodeling rather than isolated protein changes.

The most prominent pathway enrichments included ECM organization, collagen chain trimerization, assembly of collagen fibrils, collagen biosynthesis, and collagen degradation. The positive z-scores associated with these pathways indicate predicted functional activation, consistent with enhanced ECM turnover and dynamic collagen remodeling. Such coordinated regulation of matrix-related processes suggests active structural reorganization rather than passive protein accumulation, a pattern well aligned with established features of diabetic tissue remodeling.

Diabetes-associated metabolic stress, particularly hyperglycemia-driven oxidative stress and the formation of advanced glycation end products (AGEs), is known to disrupt collagen homeostasis, promoting both aberrant matrix deposition and degradation [46,55]. In contrast to the Akita model, the STZ group demonstrated activation of both integrin signaling and GP6 signaling. These pathways are centrally involved in cell–matrix and collagen-dependent signaling, where GP6 functions as a major collagen receptor and integrins regulate cell adhesion and bidirectional ECM communication. Their concurrent activation suggests enhanced matrix sensing and strengthened cell–ECM interactions rather than suppression of matrix regulatory processes. This interpretation is further supported by the enrichment of collagen-related pathways, including collagen biosynthesis, fibril assembly, and collagen degradation, collectively indicating increased ECM turnover and dynamic matrix remodeling.

Unlike the Akita dataset, which pointed toward impaired ECM regulatory activity, the STZ profile is consistent with an actively remodeling extracellular environment, potentially reflecting stress-induced structural adaptation, matrix reorganization, and collagen-driven signaling responses [44,45]. According to current models of diabetic wound pathology, chronic inflammation perpetuates activation of wound-healing signaling cascades even in the absence of effective tissue repair. Persistent pro-inflammatory cytokine signaling maintains engagement of pathways related to ECM remodeling and tissue stress responses, contributing to the apparent enrichment of the Wound Healing Signaling Pathway in the STZ group [56].

The Neutrophil Extracellular Trap (NET) Signaling Pathway exhibited a negative z-score, indicating predicted functional inhibition. NET formation represents a specialized innate immune response implicated in inflammatory and vascular injury. The predicted inhibition of NET signaling may indicate altered neutrophil-associated immune dynamics or dysregulated innate immune responses under diabetic metabolic stress [57].

The Hepatic Fibrosis/Hepatic Stellate Cell Activation pathway was enriched without a predicted activation state. In IPA, absence of a z-score reflects insufficient directional evidence, not lack of biological relevance. Enrichment of fibrosis-related pathways likely reflects shared ECM and collagen components rather than organ-specific fibrotic processes [58].

Heatmap analysis of STZ retinas revealed a striking and coordinated downregulation of α-, β-, and γ-crystallin family members, highlighting suppression of key molecular chaperones involved in oxidative stress defense and cytoskeletal stability. The tight clustering of crystallins suggests pathway-level regulation rather than isolated protein changes, indicating disruption of intrinsic neuroprotective networks in chemically induced diabetes. Additional differential expression of immune- and signaling-associated proteins further supports multifactorial remodeling of retinal homeostasis. Notably, in contrast to the Akita model, where immune-related proteins were prominently upregulated, the STZ retina exhibited dominant suppression of structural and protective proteins, suggesting model-specific mechanisms underlying diabetic retinal injury.

After applying the selection criteria, no overlapping significantly dysregulated proteins were identified between the Akita (n = 14) and STZ (n = 8) groups.

Although the Akita (n = 14) and STZ (n = 8) models shared zero individual significantly dysregulated proteins, functional pathway analysis revealed converging downstream biological processes. This lack of direct molecular overlap, coupled with shared pathway disruption, suggests that genetic and chemically induced models engage distinct nodal regulatory proteins to drive a common diabetic phenotype. Rather than targeting identical protein nodes, each model appears to alter unique components within shared functional networks, reflecting model-dependent mechanisms of cellular stress and metabolic adaptation.

Finally, a potential limitation of this study is the sample size (n = 4–6 per group), which was balanced against the high resource demands of dual-model profiling and proteomic sequencing. To mitigate this constraint and prevent false positives, we enforced stringent statistical thresholds, requiring both significant FDR control (q < 0.05) and large fold changes (|*AVG Log2 Ratio*| ≥ 1). While post-hoc power estimations indicate sufficient power to detect high-effect targets, these identified proteomic profiles represent a baseline discovery set that warrants validation in larger functional cohorts.

## 5. Conclusions

Collectively, the proteomic analysis identified numerous dysregulated proteins with potential mechanistic and therapeutic relevance in DR. LRRC58 and coronin-2A were consistently upregulated across models, supporting a pro-inflammatory retinal environment, while S100-A4 and COL4A2 increases align with angiogenic and vascular remodeling processes. In contrast, several crystallin family members were downregulated, suggesting impairment of intrinsic stress-response and neuroprotective mechanisms. Whether restoration of crystallin pathways could be therapeutically beneficial warrants further investigation.

Canonical pathway analysis showed activation of platelet-related signaling, suggesting increased platelet activity, microthrombus formation, and possible blood–retinal barrier disruption. Lipid-associated pathways were also enriched, indicating metabolic dysregulation, along with significant alterations in ECM organization and remodeling consistent with structural vascular changes in DR. Overall, these pathway-level findings provide a systems-based framework for prioritizing mechanisms and guiding targeted experimental validation.

## Figures and Tables

**Table 1:** Baseline physical and physiological characteristics of diabetic mouse models and non-diabetic controls.

Parameter	Control (WT)	STZ-Induced	Control (Ins2^+/+^)	Akita (C57BL/6J Ins2^Akita^)
Species/Background	C57BL/6J	C57BL/6J	C57BL/6J	C57BL/6J
Sex	Male	Male	Male	Male
Sample Size (n)	5	6	4	4
Age at Study Termination	~22 weeks	~22 weeks	20–22 weeks	20–22 weeks
Duration of Diabetes	N/A	16 weeks	N/A	16 weeks
Initial Body Weight (g)	24.8 ± 1.2	25.1 ± 1.1	24.5 ± 1.0	24.2 ± 1.3
Final Body Weight (g)	31.5 ± 1.8	23.4 ± 1.5	30.8 ± 1.6	22.1 ± 1.4
Fasting Blood Glucose (mg/dL)	125 ± 12	465 ± 38	130 ± 15	480 ± 42

Data are presented as mean ± SD. STZ, streptozotocin; WT, wild type; *Ins2*, insulin 2 gene; n, number of animals per group.

**Table 2:** Top 20 dysregulated proteins in the Akita retina.

Top 20 Dysregulated Proteins In Akita Group				
Name	Change	Fold Change (log^2^)	Significance (−log^10^ *p*-Value)	Manhattan Distance
Immunoglobulin kappa constant	Increased	2.702055149	2.27	4.972055149
Ig heavy chain V region MOPC 104E	Increased	1.751366679	2.09	3.841366679
Ig gamma-3 chain C region	Increased	1.381467469	2.77	4.151467469
Leucine-rich repeat-containing protein 58	Increased	1.371541334	5.31	6.681541334
Protein S100-A4	Increased	1.065146087	3.59	4.655146087
Beta-crystallin B2	Increased	1.010718512	2.81	3.820718512
Alpha-1-acid glycoprotein 1	Increased	1.001550707	3.75	4.751550707
Haptoglobin	Increased	0.971130854	4.66	5.631130854
Heat shock protein beta-1	Increased	0.956826904	3.15	4.106826904
Corticosteroid-binding globulin	Increased	0.93718034	2.59	3.52718034
Lengsin	Increased	0.822823237	2.78	3.602823237
RNA-binding protein 3	Increased	0.792825612	2.71	3.502825612
Ethanolamine-phosphate phospho-lyase	Increased	0.672939221	3.08	3.752939221
Neurogranin	Increased	0.640041784	4.89	5.530041784
Normal mucosa of esophagus-specific gene 1 protein	Increased	0.601123186	4.01	4.611123186
Keratin, type I cytoskeletal 24	Decreased	−0.964641724	3.14	4.104641724
F-box only protein 50	Decreased	−1.189502782	3.04	4.229502782
Alpha-1-antitrypsin 1-5	Decreased	−1.441426871	2.38	3.821426871
Keratin-associated protein 19-3	Decreased	−1.709205793	2.36	4.069205793
WAP four-disulfide core domain protein 12	Decreased	−2.450520366	2.51	4.960520366

The top 20 differentially expressed proteins identified in the Akita group compared to controls, ranked by Manhattan distance. For each protein, direction of change, fold change (log^2^), and statistical significance (−log^10^ *p*-value) are shown.

**Table 3:** Top 20 dysregulated proteins in the STZ retina.

Top 20 Dysregulated Proteins in STZ-Injected Group				
Name	Change	Fold Change (log^2^)	Significance (−log^10^ *p*-Value)	Manhattan Distance
Major urinary protein 20	Increased	2.066997428	3.35	5.416997428
Collagen alpha-2(IV) chain	Increased	1.822162067	1.9	3.722162067
Coronin-2A	Increased	1.444476498	2.74	4.184476498
Cytochrome c oxidase subunit 6A1, mitochondrial	Increased	0.968175459	3.13	4.098175459
Immunoglobulin heavy constant mu	Increased	0.693436694	4.6	5.293436694
Centrosomal protein kizuna	Increased	0.685611708	2.83	3.515611708
Thyroid hormone receptor alpha	Increased	0.670719217	4.43	5.100719217
Ermin	Increased	0.668535833	3.97	4.638535833
Protein kintoun	Increased	0.633080177	7.67	8.303080177
Chloride intracellular channel protein 6	Increased	0.617985276	4.66	5.277985276
Beta-crystallin B2	Decreased	−0.607681561	6.15	6.757681561
Beta-crystallin A1	Decreased	−0.627056321	8.74	9.367056321
Beta-crystallin A4	Decreased	−0.641359568	11.7	12.34135957
Beta-crystallin A2	Decreased	−0.776108996	8.44	9.216108996
Beta-crystallin B1	Decreased	−0.783809074	11.1	11.88380907
Gamma-crystallin S	Decreased	−0.802267743	9.08	9.882267743
Gamma-crystallin D	Decreased	−0.94362112	11.8	12.74362112
Gamma-crystallin C	Decreased	−1.058565814	8.18	9.238565814
Nuclear factor NF-kappa-B p100 subunit	Decreased	−1.180978714	2.46	3.640978714
Gamma-crystallin B	Decreased	−1.272391855	8.91	10.18239186

The top 20 differentially expressed proteins identified in the STZ group compared to controls, ranked by Manhattan distance. For each protein, direction of change, fold change, and statistical significance (−log^10^ *p*-value) are shown.

## Data Availability

The mass spectrometry proteomics data have been deposited to the ProteomeXchange Consortium (https://www.proteomexchange.org/, accessed on 22 October 2025) via the PRIDE (https://www.ebi.ac.uk/pride/, accessed on 22 October 2025) partner repository with the dataset identifier PXD069751.

## Supplementary Materials

The following supporting information can be downloaded at: https://www.mdpi.com/article/10.3390/biom16091314/s1, Figure S1: The original western blot images of βB2 crystallin expression in control and Akita diabetic retina; Figure S2: The original western blot images of βB2 Crystallin in Proteomic Samples blot; Table S1: Differentially expressed proteins in Akita and STZ diabetic mouse models.

## Abbreviations

DR	Diabetic retinopathy
STZ	Streptozotocin
T1DM	Type 1 diabetes mellitus
BRB	Blood-retinal barrier
DME	Diabetic macular edema
PDR	Proliferative diabetic retinopathy
VEGF	Vascular endothelial growth factor
Cry	Crystallin
IgG3	Immunoglobulin gamma-3 chain C region
LRRC58	Leucine-rich repeat-containing protein 58
CDO1	Cysteine dioxygenase 1 (complex targeting cysteine dioxygenase 1)
RBM3	RNA-binding protein 3
NMES1	Normal mucosa of esophagus-specific gene 1
FBXO50	F-box only protein 50
KRT24	Keratin, type I cytoskeletal 24
KRTAP19-3	Keratin-associated protein 19-3
WFDC12	Whey acidic protein four-disulfide core domain protein 12
TLR	Toll-like receptors
LXR/RXR	Liver X receptor/retinoid X receptor
DHCR24	24-dehydrocholesterol reductase
MUP20	Major urinary protein 20
COL4A2	Collagen alpha-2(IV) chain
COX6A1	Cytochrome c oxidase subunit 6A1
IGHM	Immunoglobulin heavy constant mu
THRA	Thyroid hormone receptor alpha
CLICs	Chloride intracellular channels
NFKB2	NF-κB p100 subunit
AGEs	Advanced glycation end products
ECM	Extracellular matrix
NET	Neutrophil extracellular traps
