# Supplementary material for: Proteomic Analysis of Retinas from Two Different Type-1 Diabetic Models
Source: Biomolecules. 2026 Sep 10;16(9):1314. doi: 10.3390/biom16091314 (PMC13604719; doi:10.3390/biom16091314)

Table S1. Differentially expressed proteins in Akita and STZ diabetic mouse models.

Summary of significantly dysregulated proteins identified in male Akita (n=14) and STZ-induced (n=8) diabetic models compared to their respective age-matched non-diabetic controls. Thresholds for significance were set at p < 0.05, q < 0.05 (False Discovery Rate), p < 0.05, q < 0.05, and ∣AVG Log2 Ratio∣ ≥ 1((≥2-fold change). Positive AVG Log2 Ratio values indicate upregulation in diabetic tissues; negative values indicate downregulation.

Akita (n=14)						
**UniProtIds**	Genes	ProteinDescriptions	ProteinNames	AVG Log_2 Ratio	Pvalue	Qvalue
**Q3UGP9**	Lrrc58	Leucine-rich repeat-containing protein 58	LRC58_MOUSE	1.371541	4.89E-06	0.002837
**Q60590**	Orm1	Alpha-1-acid glycoprotein 1	A1AG1_MOUSE	1.001551	0.000178	0.012571
**P07091**	S100a4	Protein S100-A4	S10A4_MOUSE	1.065146	0.000256	0.014352
**G3X9C2**	Nccrp1	F-box only protein 50	FBX50_MOUSE	-1.1895	0.000917	0.024425
**P62696**	Crybb2	Beta-crystallin B2	CRBB2_MOUSE	1.010719	0.001554	0.029799
**P03987**	NaN	Ig gamma-3 chain C region	IGHG3_MOUSE	1.381467	0.001686	0.030716
**Q9JHY3**	Wfdc12	WAP four-disulfide core domain protein 12	WFD12_MOUSE	-2.45052	0.003079	0.039266
**P14069**	S100a6	Protein S100-A6	S10A6_MOUSE	-1.07909	0.003931	0.042344
**Q00898**	Serpina1e	Alpha-1-antitrypsin 1-5	A1AT5_MOUSE	-1.44143	0.004207	0.043642
**Q925H6**	Krtap19-3	Keratin-associated protein 19-3	KR193_MOUSE	-1.70921	0.004331	0.04412
**Q9WUA5**	Epm2a	Laforin	EPM2A_MOUSE	1.003746	0.004524	0.044865
**P01837**	Igkc	Immunoglobulin kappa constant	IGKC_MOUSE	2.702055	0.005369	0.047658
**O08999**	Ltbp2	Latent-transforming growth factor beta-binding protein 2	LTBP2_MOUSE	-1.13286	0.005452	0.04782
**Q9JLJ5**	Elovl1	Elongation of very long chain fatty acids protein 1	ELOV1_MOUSE	-1.23291	0.005743	0.048857
**STZ (n=8)**						
**UniProtIds**	Genes	ProteinDescriptions	ProteinNames	AVG Log_2 Ratio	Pvalue	Qvalue
**P04344**	Crygb	Gamma-crystallin B	CRGB_MOUSE	-1.27239	1.23E-09	8.27E-07
**Q61597**	Crygc	Gamma-crystallin C	CRGC_MOUSE	-1.05857	6.68E-09	3.50E-06
**Q5FW60**	Mup20	Major urinary protein 20	MUP20_MOUSE	2.066997	0.000447	0.007767
**Q8C0P5**	Coro2a	Coronin-2A	COR2A_MOUSE	1.444476	0.001807	0.016088
**Q9WTK5**	Nfkb2	Nuclear factor NF-kappa-B p100 subunit	NFKB2_MOUSE	-1.18098	0.003443	0.022569
**P01868;P01869**	Ighg1	Ig gamma-1 chain C region secreted form;Ig gamma-1 chain C region, membrane-bound form	IGHG1_MOUSE;IGH1M_MOUSE	-1.14466	0.007188	0.032824
**Q9D6L8**	Ppil3	Peptidyl-prolyl cis-trans isomerase-like 3	PPIL3_MOUSE	1.361027	0.012258	0.043279
**P08122**	Col4a2	Collagen alpha-2(IV) chain	CO4A2_MOUSE	1.822162	0.012606	0.043895

Figure S1. The original western blot images of βB2 crystallin expression in control and Akita diabetic retina

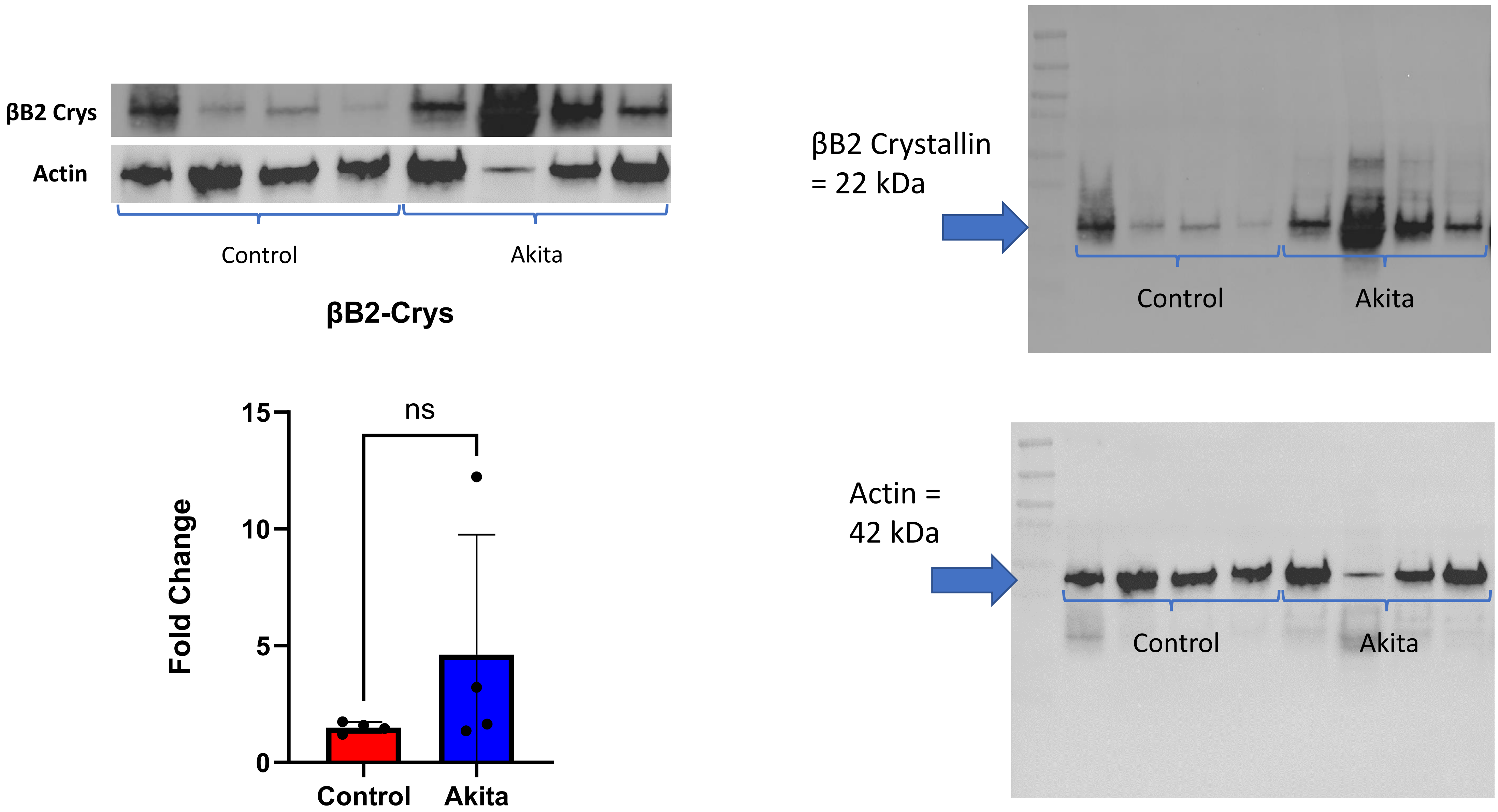

Figure S2 The original western blot images of βB2 Crystallin in Proteomic Samples blot

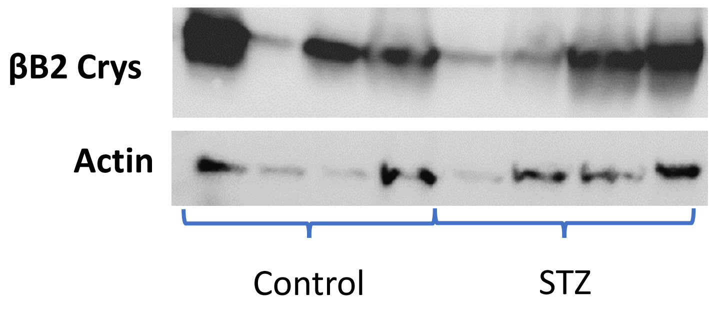

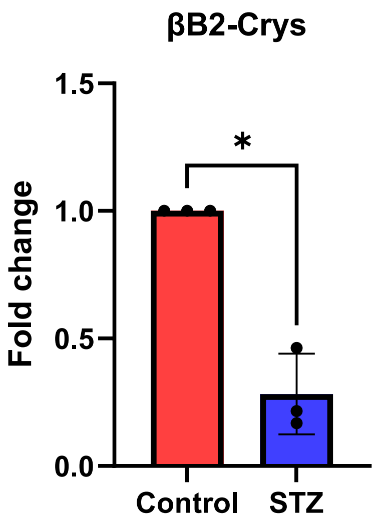

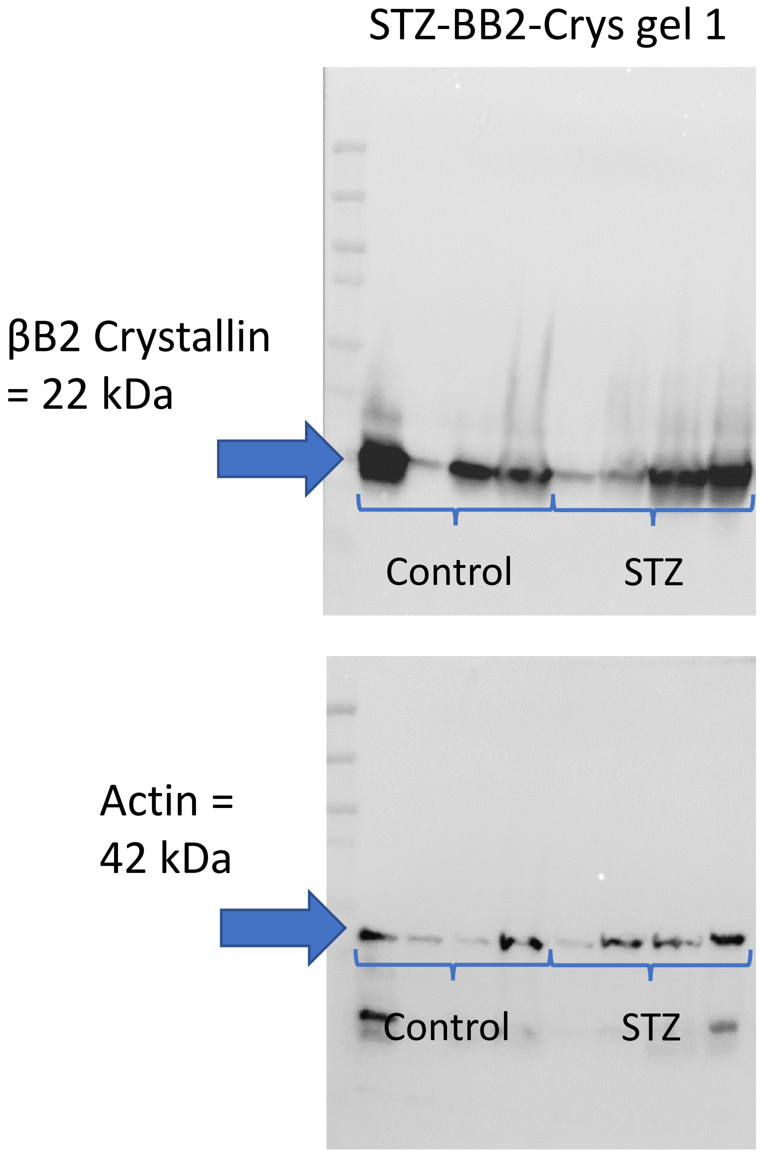

STZ-BB2-Crys gel 2

βB2 Crystallin
= 22 kDa

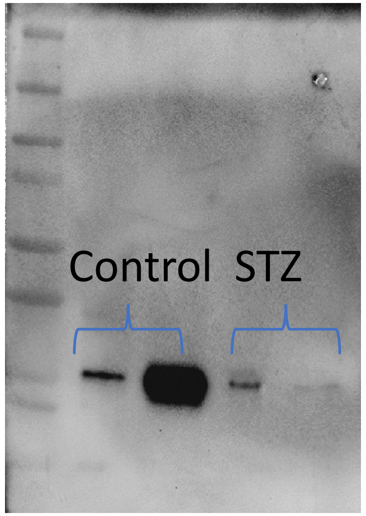

GAPDH
37 kDa

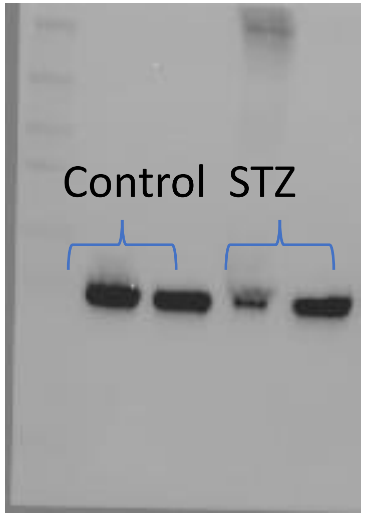

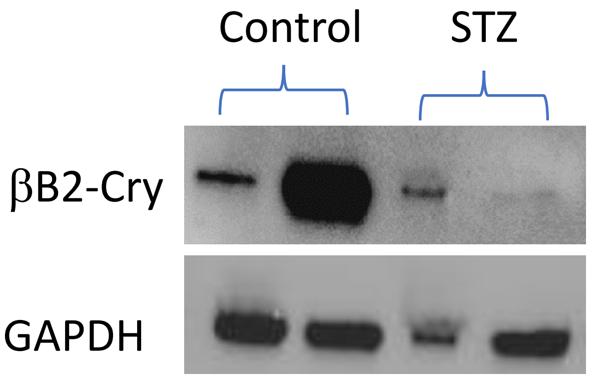

Actin =
42 kDa

STZ-BB2-Crys gel 3

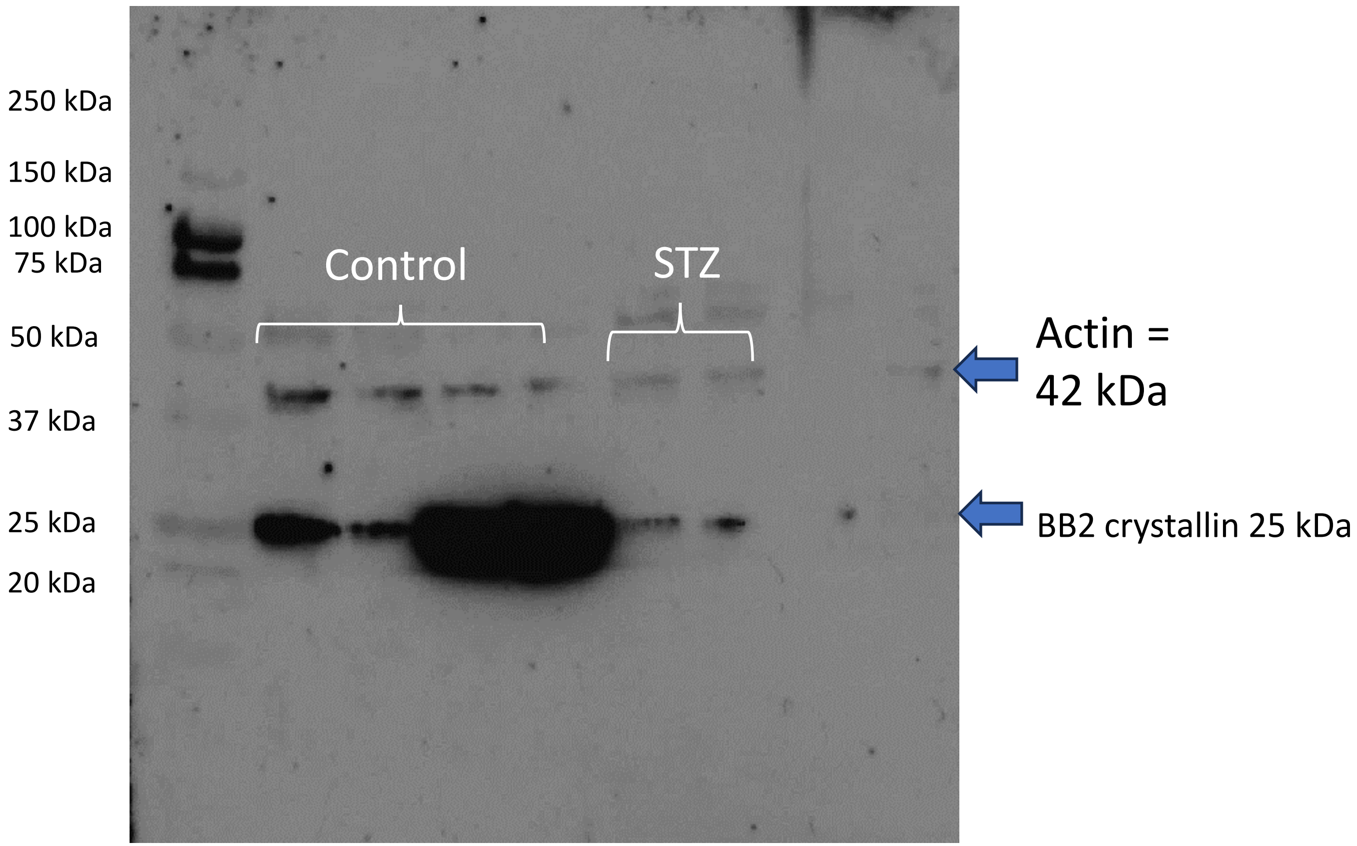

Supplement: Supplementary file 1 [file biomolecules-16-01314-s001.zip › biomolecules-4401108-supplementary.pdf]
